# The complex interaction between mammalian herbivores, climate, soil and fire has shaped the evolution and distribution of plant spinescence across biogeographical realms

**DOI:** 10.1111/nph.71376

**Published:** 2026-07-22

**Authors:** Rachel Souza Ferreira, Jens J. Ringelberg, Colin Hughes, Eduardo Arlé, Friederike J. R. Wölke, Kyle W. Tomlinson, Renske E. Onstein

**Affiliations:** ^1^ German Center for Integrative Biodiversity Research (iDiv) Halle – Jena – Leipzig Puschstrasse 4 Leipzig 04103 Germany; ^2^ Leipzig University Johannisallee 21–23 Leipzig D‐04103 Germany; ^3^ Naturalis Biodiversity Center Darwinweg 2 Leiden 2333 CR the Netherlands; ^4^ Biosystematics Group Wageningen University and Research Radix Building 107, Droevendaalsesteeg 1 Wageningen 6708 PB the Netherlands; ^5^ Department of Systematic and Evolutionary Botany University of Zurich Zollikerstrasse 107 Zurich CH 8008 Switzerland; ^6^ School of Zoology, George S. Wise Faculty of Life Sciences Tel Aviv University Tel Aviv 6997801 Israel; ^7^ Department of Ecology, Environment and Plant Sciences Stockholm University Stockholm SE‐106 91 Sweden; ^8^ Center for Integrative Conservation & Yunnan Key Laboratory for Conservation of Tropical Rainforests and Asian Elephants, Xishuangbanna Tropical Botanical Garden Mengla Yunnan 666303 China; ^9^ Center of Conservation Biology Core Botanical Gardens, Chinese Academy of Sciences Menglun Yunnan 666303 China; ^10^ Leiden University, Institute of Biology Leiden Sylviusweg 72 Leiden 2333 BE the Netherlands

**Keywords:** arms race, defence trait, Fabaceae, herbivory, Mimoseae, plant–herbivore interaction

## Abstract

- The evolutionary arms race between plants and herbivores led to numerous plant adaptations, including spinescence. However, the balance between herbivory and abiotic conditions in the evolution of spinescence remains unclear.
- We integrated phylogenetic, geographic, and trait data for 2686 species of an ecologically diverse and spinescent pantropical lineage, mimosoid legumes, with distribution data on 368 extant and extinct mammalian herbivores ≥ 10 kg. Using structural equation models, we assessed how herbivores, climate, soil, and fire directly and indirectly affected the proportion of spinescent mimosoids across global and continental assemblages.
- Models incorporating extinct herbivore assemblages explained more variation in the proportion of spiny species than those based solely on extant herbivores. Dry‐season length and soil pH increased spinescence both directly and indirectly through their effects on herbivore richness. These abiotic effects exceeded herbivore effects at continental scales. Fire influenced spinescence only indirectly via its positive relationship with herbivore richness. Finally, spinescence evolved repeatedly across mimosoids from *c.* 35 million years ago, pre‐dating the Miocene savanna expansion.
- Our study suggests that past herbivore communities have left a lasting imprint on present‐day plant defence patterns and that long‐term climatic transitions and the emergence of open, herbivore‐rich landscapes played crucial roles in the evolution and distribution of spinescence.

## Graphical abstract

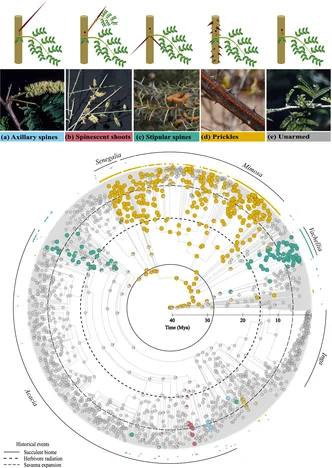

## Introduction

The consumption of plant tissues by herbivores has driven an evolutionary arms race, leading to a diverse range of defence mechanisms and adaptations, expansions in ecological space, and adaptive radiations in plants and herbivores (Dawkins & Krebs, 1979; Endara *et al*., 2017; Perkovich & Ward, 2022). Herbivorous mammals, for example, exhibit diverse feeding behaviours based on the plant parts they consume: grazers primarily consume grasses, while browsers feed on the leaves, flowers and fruits of woody plants, and mixed feeders consume both grasses and woody vegetation (Owen‐Smith, 1997). In response, plants have evolved a variety of defence traits to protect themselves from herbivory, including chemical (e.g. high‐tannin levels), structural (e.g. spinescence), and symbiotic traits (e.g. bodyguarding ants, such as those associated with *Vachellia* species in Central America and African savannas) (War *et al*., 2012; Palmer, 2023). Among these traits, structural defences, such as spines, spinescent shoots, and prickles, are particularly effective against large‐bodied herbivores (i.e. herbivores ≥ 10 kg body mass) functioning as barriers that injure the animals' mouths during feeding (Dickison, 2000; Valverde *et al*., 2001).

Although spinescence is generally associated with the presence of herbivores (Cooper & Owen‐Smith, 1986; Tomlinson *et al*., 2025), other factors may also influence its occurrence. First, spinescence may be particularly advantageous under certain climatic conditions. For example, in areas with low precipitation and prolonged dry seasons, plants tend to allocate more resources to defence mechanisms, as water scarcity limits photosynthesis and growth, increasing vulnerability to herbivory (Grubb, 1992; Vilela *et al*., 2012; Armani *et al*., 2019). Second, soil resource availability can mediate herbivory, as variation in soil fertility influences leaf nutrient concentrations and associated chemical traits, thereby affecting plant palatability to herbivores (Bell, 1982; Tomlinson *et al*., 2016, 2025; Wigley *et al*., 2018; Dantas & Pausas, 2022; Wang *et al*., 2023). Third, extinct herbivores may have left an imprint (‘legacy effect’) on the distribution of spinescence in ecosystems today (Pu *et al*., 2025). For example, spiny thicket areas of southern Madagascar and the Caatinga in north‐east Brazil likely had historically rich large‐bodied herbivore faunas until the Late Pleistocene (*c*. 10 000 yr ago) but are now relatively poor in mammalian herbivores, with the notable exception of introduced livestock (Mooney *et al*., 1995). Spinescence in these systems may thus represent adaptations to browsing by now extinct large herbivores, making them evolutionary anachronisms (Janis, 2002).

Nevertheless, spinescent plants remain common in places with high densities of extant large herbivores, such as fire‐prone African savannas (Hempson *et al*., 2015; Charles‐Dominique *et al*., 2016). Herbivores regulate fuel availability by altering plant biomass, thereby influencing fire intensity and frequency. Fire, in turn, kills woody plants and promotes resprouting in both woody and herbaceous species, influencing herbivore distribution and feeding behaviour across landscapes (van Langevelde *et al*., 2003; Pringle *et al*., 2015; Archibald & Hempson, 2016; Rouet‐Leduc *et al*., 2021). Together, fire and herbivores maintain open vegetation, such as savannas (van Langevelde *et al*., 2003). Through this feedback loop, variation in fire regimes may reinforce herbivore‐driven selection for spinescence.

Most spinescent woody plant lineages in African savannas diversified from the early Miocene onwards (Charles‐Dominique *et al*., 2016), possibly in response to increasing browsing pressures from large herbivores (Staver *et al*., 2012; Charles‐Dominique *et al*., 2016; Dantas & Pausas, 2022). This diversification also coincided with the rise of bovids and the geographical expansion of African savannas (Beerling & Osborne, 2006; Charles‐Dominique *et al*., 2016), creating ecological opportunities for spinescent plants. However, spinescence probably originated much earlier, dating back to the Eocene (Zhang *et al*., 2022) or even the Cretaceous in some lineages (e.g. in palms (Arecaceae); Onstein *et al*., 2022).

The drivers of the origin, diversification, and distribution of spinescent lineages remain unclear, but likely resulted from complex interactions between large herbivores, climate, soil, and fire. Notably, Africa and the Americas present striking contrasts in large herbivore populations (Owen‐Smith, 1987, 2013), providing a comparative framework to understand the relative importance of historical and contemporary drivers of spinescence. Although South America and Africa had historically similar levels of herbivore richness (Owen‐Smith, 2013), South America experienced more severe megafauna extinctions during the Pleistocene, losing *c*. 80 species (38.65%) of herbivores over 10 kg, while Africa lost only 27 species (17.65%) (Stuart, 2015; Faurby *et al*., 2018). However, declines in distribution ranges are evident in both regions. Such local, global, or functional extinctions may have substantially changed ecosystems, potentially increasing woody cover in South American savannas (Doughty *et al*., 2016) and promoting more frequent wildfires due to the accumulation of plant matter (Galetti, 2004), or even driving transitions from savanna to forest. Past interactions with megafauna may also explain continental differences in plant traits, such as higher wood densities, taller plants, more spines (Dantas & Pausas, 2013, 2020, 2022), and larger fruits (Mack, 1993; Wölke *et al*., 2023) in African savannas compared with the Brazilian Cerrado, the largest South American savanna. While Africa was dominated by intermediate size grazers and tall browsers such as elephants and giraffes, South America's Late Pleistocene megafauna was dominated by mixed feeders and very large grazers and browsers (e.g. notoungulates, equids, and giant ground sloths; Owen‐Smith, 2013). These differences in the composition and functional roles of large‐herbivore assemblages likely generated different levels of herbivore pressure on plants, shaping vegetation structure and the evolution of spinescent lineages.

Previous studies have documented continental patterns in spinescence (Charles‐Dominique *et al*., 2016; Dantas & Pausas, 2022; Tomlinson *et al*., 2025), identified climatic and soil effects on herbivore distributions (Hempson *et al*., 2015; Dantas & Pausas, 2022), and reconstructed the evolutionary history of spinescence within and across lineages (Charles‐Dominique *et al*., 2016; Anest *et al*., 2024). Here, we present a novel global, phylogenetically controlled dataset that integrates extant and Late Quaternary large‐bodied herbivorous mammals to test how herbivores, climate, soils, and fire jointly shaped the distribution of spinescence, and to compare the contrasting (large) faunal histories of Africa and the Americas.

We use mimosoid legumes (tribe Mimoseae, formerly subfamily Mimosoideae) (Bruneau *et al*., 2024), a pantropical clade of *c*. 3500 species of trees, lianas, shrubs, and geoxyles, as a study system. Mimosoids form ecologically important or even dominant elements in all major lowland tropical ecosystems, including rainforests, savannas, and seasonally dry forests (Koenen *et al*., 2020; Ringelberg *et al*., 2023). Importantly, mimosoids have evolved a wide range of defence traits against large herbivores, including thorns, spines, and prickles, which are morphologically nonhomologous (i.e. derived from different plant structures) but functionally convergent in deterring herbivory. Spinescence evolved independently multiple times throughout mimosoid evolution (Ringelberg *et al*., 2022) likely contributing to the diversification of mimosoids across the tropics, and resulting in radiations of spinescent lineages in different regions, such as *Vachellia* and *Senegalia* in the American and African drylands (Greve *et al*., 2012) and mesquites (*Neltuma* and *Strombocarpa*, formerly *Prosopis*) in the seasonally dry tropical scrublands of the succulent biome in the Americas (*sensu* Schrire *et al*., 2005). Several species of Australian mimosoids (the vast majority of which are in the genus *Acacia*) have sharpened or stiffened leaf tips that may also function as defence (Tomlinson *et al*., 2025). However, in this study, we concentrate on prickles, axillary spines, spinescent shoots, and stipular spines (hereafter ‘spinescence’), which serve as mechanical defences that protect leaves from herbivore consumption. We present a newly assembled spinescence trait dataset for 2686 mimosoid species (76.7% of all species) and combine this with extensive species distribution data and a time‐calibrated phylogeny to investigate the evolution and distribution of spinescence across space and time. Given the complex interplay of factors potentially influencing spinescence, our study aimed to unravel the interaction between large herbivores, climate, soil, and fire by testing the following hypotheses:

Hypothesis 1 (H1) *Biotic drivers of spinescence: Species richness of extant and extinct large herbivores determines global and regional variation in the proportion of spinescent mimosoid species across assemblages, because, to effectively deter herbivory, these structures should have adapted and co‐evolved with the feeding behaviour and diversity of herbivores (Burns,* *2014* *; Perkovich & Ward,* *2022* *). Extinct large herbivores may be particularly important for explaining the distribution of spinescence in regions that lost most large herbivores in the Pleistocene (i.e. the Americas), reflecting a legacy effect (Stuart,* *2015* *; Pu et al.,* *2025* *).*

Hypothesis 2 (H2) *Abiotic drivers of spinescence: Drought, temperature, soil fertility, and fire influence spinescence directly and/or indirectly given their effect on plant growth rate and thus ability to recover under mammalian herbivory pressure. Accordingly, spinescence is expected to be more prevalent where biomass loss is more costly and herbivory pressure is stronger. Specially, towards more arid climates, continuous periods of low rainfall (dry seasons) increase in length, reducing the plant growth period thereby increasing the cost of losing biomass (e.g. leaves). Concurrently, drought influences herbivory pressure and herbivore species richness through its effects on vegetation structure and productivity (Hempson et al.,* *2015* *). Jointly, drought and herbivores increase the selection for plant defence (Tomlinson et al.,* *2025* *). Similarly, temperature directly influences plant growth and development, increasing the cost of biomass lost to herbivory in cooler climates, in turn selecting for spinescence. Soil characteristics also modulate these dynamics: more fertile, nutrient‐rich, slightly acidic, or alkaline soils (i.e. medium to high pH) tend to increase leaf nutrient concentrations that attract more herbivores, increasing herbivory pressure and strengthening selection for spinescence (Bell,* *1982* *; Dantas & Pausas,* *2022* *). Finally, fire and herbivores interact through plant biomass feedbacks: herbivores reduce fuel loads while fire suppresses woody plants, reducing food availability for herbivores and herbivore‐driven selection for spines.*

## Materials and Methods

### Spatial data resolution and assembly

To test whether global spinescence patterns are related to interactions with large herbivores and other factors, we quantified spinescent species proportions, extant and extinct mammalian herbivore richness, bioclimatic and soil variables, and mean burn area across the global distribution of mimosoids. We used an equal‐area grid (Behrmann projection) with *c*. 100‐km resolution (≈1° at the equator). This grid was used to extract species ranges and environmental variables, assemble presence–absence data, and calculate mean environmental conditions per cell.

### Mimosoid data

Trait data on herbivore defences were compiled using the mimosoid species checklist from Ringelberg *et al*. (2023), with generic updates following Bruneau *et al*. (2024). The mimosoid clade, formerly known as subfamily Mimosoideae, comprises *c*. 3500 species across > 100 genera. Trait data were compiled for 2686 species (76.7%) spanning all genera. Spinescence presence and type were classified (Bell & Bryan, 2008) by assigning each species to one of five spinescence states: prickles, stipular spines, axillary spines, spinescent shoots, and unarmed (Supporting Information Table S1). Sources used to score spinescence for each taxon including global and regional floras, monographs, species descriptions, and herbarium specimens are listed in Notes S1.

Proportion of spiny species per grid cell was calculated as the number of spinescent species divided by the total number of mimosoid species. Occurrence data followed Ringelberg *et al*. (2023), based on 444 471 curated records from GBIF, DryFlor, SEINet, and regional sources after taxonomic updating and filtering of cultivated, centroid, marine, and non‐native records. The study region was defined by the global distribution of mimosoid species rather than by fixed latitudinal limits. Although predominantly pantropical, a small number of mimosoid species extend into warm temperate regions, which were therefore retained in the analyses. For assemblage and structural equation model (SEM) analyses, spinescence was treated as a binary functional umbrella trait. However, because spinescence types are derived from different plant parts (stipules, axillary buds, shoots, and epidermis) and are thus nonhomologous, they were treated as separate traits for phylogenetic character reconstruction.

### Environmental data

Mean temperature (BIO1) was obtained from CHELSA 1.2 (Karger *et al*., 2017). Dry season length was calculated as the number of consecutive months with rainfall < 100 mm from CHELSA 1.2. Mean burn area, calculated as fires per km^2^ of natural area, was derived from MODIS MCD14ML active‐fire detections (2000–2019) (Giglio *et al*., 2016), retaining only high‐confidence vegetation fires (≥ 95%) in natural areas (≥ 90% natural vegetation cover) (Defourny, 2017). Soil pH was calculated as the mean of the values at 0.05 and 2 m depth from SoilGrids 2.0 (Hengl *et al*., 2014) and used as a proxy for soil fertility, although nutrient availability varies among elements and is further influenced by climate (Viani *et al*., 2014; Dantas & Pausas, 2022; Hartemink & Barrow, 2023).

### Extant herbivore data

Current ranges of 4858 extant mammal species were obtained from PHYLACINE (Faurby *et al*., 2018), focusing on areas where species were classified as extant or native by the International Union for Conservation of Nature (IUCN, 2022 v.2016–3). Herbivores were defined as species with > 95% plant diets according to PHYLACINE (Faurby *et al*., 2018). Additionally, we identified grazer and browser species through the MammalDiet database (Kissling *et al*., 2014). We explicitly excluded species that feed exclusively on grasses, also known as ‘exclusive grazers’. Instead, our focus was directed towards species that consume leaves of woody plants, commonly referred to as ‘browsers’ or ‘mixed feeders’, given their potential interaction with mimosoid plants. In addition, we only included species with body mass equal to or > 10 kg, resulting in a dataset of 215 species, following the large mammal definition from Sandom *et al*. (2014), as structural defences such as spines are less effective at deterring small‐bodied herbivores. To visualize the overlap between herbivores and mimosoid species, we delineated their ranges using an equal‐area grid and ‘*relate*’ function in the terra R package (Hijmans, 2023).

### Extinct herbivore data

We derived the probable past distribution of most Late Quaternary extinct (153 species) and extant (215 species) medium‐ and large‐bodied terrestrial mammalian herbivore species based on the ‘present natural’ range in PHYLACINE (Faurby *et al*., 2018). This scenario is based on the distribution of extant species that coexisted with these extinct species according to fossil records. It considers an extinct species to have been present in a particular grid cell if at least 50% of the extant species found in coexistence with it in fossil records were also predicted to occupy that cell before anthropogenic changes. This method is based on the assumption that extinct and extant species that shared habitats likely had similar ecological requirements. Thus, by mapping the prehuman distribution of coexisting extant species, the historical ranges of their extinct counterparts can be inferred (Faurby *et al*., 2018). To explore the potential influence of large herbivores on plant defence, we included only species with diets comprising at least 95% of plants and body mass equal to or > 10 kg, as inferred using PHYLACINE (Faurby *et al*., 2018). The extinct taxa were matched to HerbiTraits v.1.2 (Lundgren *et al*., 2021), and guilds were standardized (browser, mixed‐feeder, grazer). We excluded obligate grazers and retained browsers and mixed feeders (Table S2).

### Structural equation models

Direct and indirect effects of the distribution of large herbivores, climate, soil, and fire on the proportion of spinescent mimosoid species (Table 1) were tested using structural equation models (SEMs) in ‘lavaan’ (Rosseel, 2012). Before fitting SEMs, we evaluated pairwise correlations among all candidate predictors, including herbivore richness, climate, soil, and fire (Fig. S1). This analysis revealed strong collinearity between soil pH and length of the dry season (*r* = 0.86). Because this degree of collinearity prevents reliable estimation of independent effects, soil pH and dry‐season length were not included in the same SEM. Furthermore, herbivore richness was classified under two scenarios: extant and present‐natural (hereafter ‘extinct’ herbivore richness). We therefore constructed four separate models, one extant, and one extinct model for each environmental configuration (soil pH or dry‐season length). Mean burn area was log‐transformed to reduce skewness, and all variables were rescaled to the 0–1 range to allow direct comparison of effect sizes.

**Table 1:** Detailed description of variables used in the structural equation models to explain the distribution of spinescence in mimosoid legumes.

Variable	Description
Proportional richness of spinescent species	The number of spinescent mimosoid species divided by the total number of mimosoid species in a given grid cell
Extant herbivore richness	Number of mammalian herbivore species > 10 kg in their current ranges from the PHYLACINE database (Faurby *et al*., 2018)
Extinct herbivore richness	Number of mammalian herbivore species > 10 kg in the present natural scenario from the PHYLACINE database (Faurby *et al*., 2018)
Length of dry season	Consecutive months with rainfall < 100 mm/month from CHELSA, spanning 1979 to 2010 (Karger *et al*., 2017) Index from (Ringelberg *et al*., 2020)
Mean burn area	Mean annual density of fires per km^2^ on natural areas from 2000 to 2019 (Giglio *et al*., 2016; Defourny, 2017)
Mean annual temperature	Mean annual temperature from CHELSA, spanning 1979 to 2013 (Karger *et al*., 2017)
Soil pH	Soil pH (pH*10) measured in water, spanning 1960 to 2020 (Hengl *et al*., 2014)

Base SEMs included direct effects of herbivore richness on spinescence, as well as effects of temperature, length of dry season, soil, and fire (Tables S3–S5a–d). We focussed on grid cells containing data for more than three mimosoid species, to ensure robust and reliable causal inferences. To assess the sensitivity of this cut‐off, we also ran the global SEMs on cells with one and five or more mimosoid species, and results remained qualitatively similar across cut‐off values (Tables S6, S7a–d). Model fit was evaluated using χ^2^ (*P* > 0.05), CFI > 0.90, and RMSEA < 0.05 (Grace *et al*., 2012). We fitted a base model and sequentially removed nonsignificant model terms until all remaining terms were significant (at *P* < 0.05). SEMs were run globally and separately for the Americas and Africa. Caribbean islands, Madagascar, and Réunion were excluded due to distinct herbivore extinction histories. We did not run models for Australia or Asia, as the proportion of spinescent mimosoids in these realms is low (but see the Discussion section).

### Spatial autocorrelation and randomizations

To account for the impact of spatial autocorrelation on our results, given the potential similarity of signals between adjacent grids, we employed simultaneous autoregressive (SAR) models. SAR models incorporate spatial autocorrelation in model residuals. The spatial‐weights matrix was constructed by identifying the maximum neighbour distance (1113.171 km) needed to ensure each cell had at least one adjacent cell, implemented with the ncf R package (Bjornstad & Cai, 2022) and then calculating weights and Moran's *I* values with the spdep R package (Bivand *et al*., 2024). We assessed spatial autocorrelation in observed spinescence distributions and in model residuals using Moran's *I* and spatial residual maps for representative global models based on the direct drivers of the proportion of spinescent mimosoids as detected in the SEM (i.e. dry‐season length or soil pH, and for extant and extinct herbivore scenarios) (Fig. S2).

Furthermore, to evaluate whether the observed effects of herbivore richness on the global distribution of spinescence could arise from confounding correlations, we performed a randomization study. We repeated the SEM after randomizing the presence and absence of spinescence across mimosoid species 1000 times, while maintaining total mimosoid species richness within each grid cell, and recalculated the proportion of (randomized) spinescent species per cell. We then compared the observed standardized coefficients of extant and extinct herbivore richness from the empirical SEM with the 1000 simulated coefficients. Under the null expectation, herbivore richness should not predict the spatial distribution of randomized spinescence, allowing us to test whether our empirical relationships exceed those expected from confounding structure alone.

### Phylogenetic ancestral state reconstruction of spinescence types

To evaluate whether the evolution of spinescence is associated with large herbivorous mammals and/or abiotic factors, we assessed whether spinescent mimosoid lineages evolved concurrently with the diversification of large herbivores, the spread of African savannas, or the expansion of the succulent biome (*sensu* Schrire *et al*., 2005). To this end, we conducted ancestral state reconstructions of spinescence types on the time‐calibrated mimosoid phylogenetic tree of Ringelberg *et al*. (2023). This phylogeny includes 1860 mimosoid species (58% of total mimosoids). The backbone topology was constructed using DNA sequence data generated through targeted enrichment of 997 nuclear genes (Koenen *et al*., 2020), and combined with 15 species‐level phylogenies (Ringelberg *et al*., 2023). We classified mimosoid species based on the presence or absence of four developmentally nonhomologous spinescence types (axillary spines, prickles, stipular spines, and spinescent shoots). Species lacking all four types were classified as ‘unarmed’. Species bearing spinescent leaf tips or margins were also considered ‘unarmed’, as leaf‐tip/margin spines were not included in our classification and occur in relatively few mimosoids.

We estimated ancestral state probabilities at all internal nodes using maximum likelihood as implemented in the ‘*ancr’* function in the phytools R package (Revell, 2011). We did this independently for each spinescence type, given their nonhomology, and subsequently combined these separate reconstructions. For each reconstruction, we fitted both Equal Rates (ER) and All Rates Different (ARD) transition models, conducting 20 independent optimization runs per model with random starting values to avoid local likelihood maxima. During each run, we applied log scaling to improve numerical stability. Model performance was evaluated across runs, and model fits were compared using log‐likelihood, analysis of variance (ANOVA) and the Akaike Information Criterion (AIC). The best‐supported model was retained for interpretation. We considered well‐supported ancestral states only when their posterior probability was ≥ 0.75; nodes below this threshold were treated as uncertain.

## Results

### Global patterns of spinescent species distribution

**Fig. 1:**
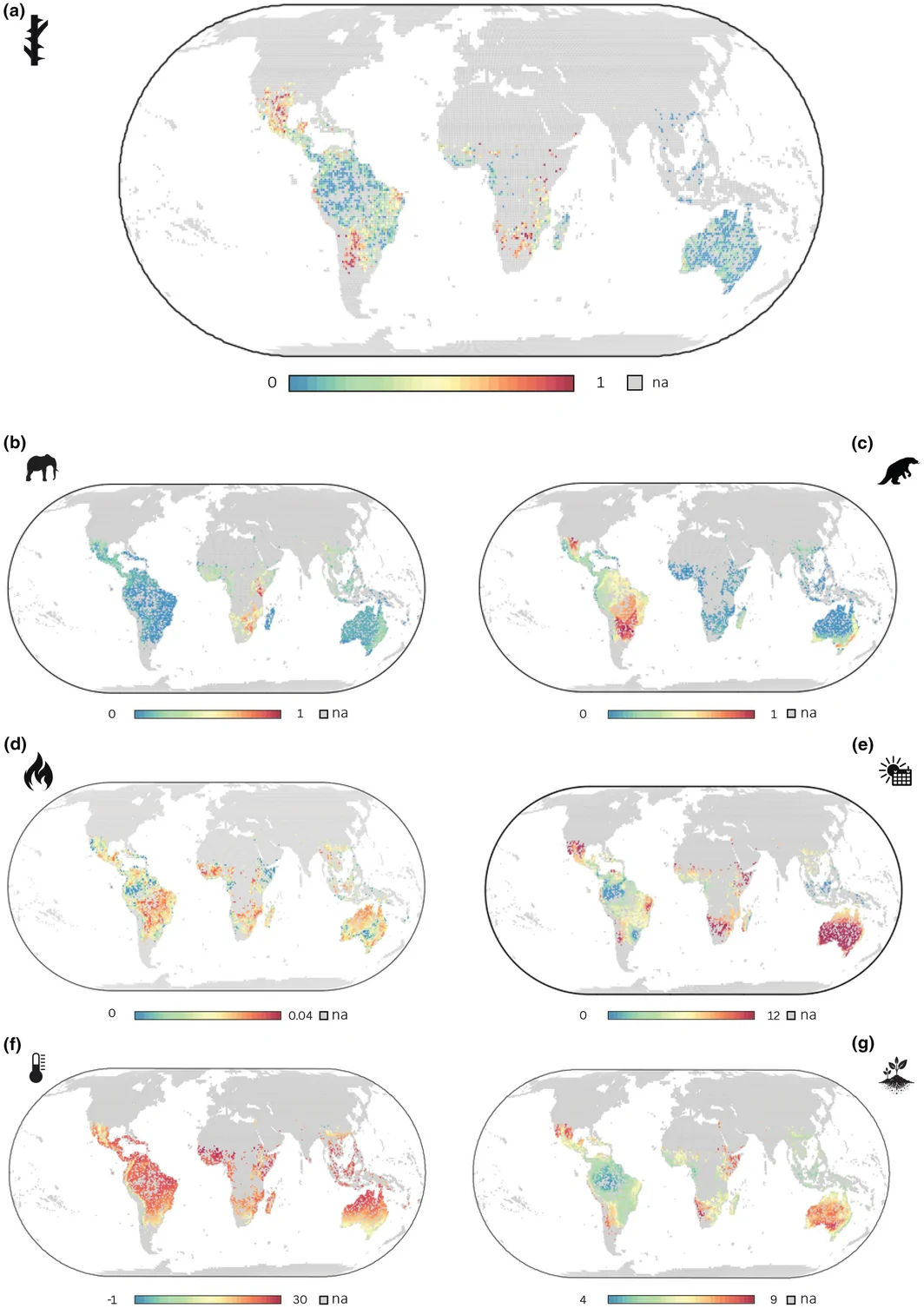
Geographical distribution of spinescence, large herbivores, and environmental factors across global mimosoid assemblages. Only grid cells (100 × 100 km; ≈1° at the equator) with > 3 mimosoid species are shown. (a) Proportion of mimosoid species bearing spines (axillary spines, prickles, stipular spines, or spinescent shoots) (assemblages range from 3 to 33 spinescent species). (b) Extant large herbivore richness (≥ 10 kg) (scaled between 0 and 1; from 0 to 18 species). (c) Extinct large herbivore richness (≥ 10 kg) (scaled between 0 and 1; from 0 to 34 species). (d) Mean burn area per year (from 2000 to 2019). (e) Length of dry season in months (from 1979 to 2010). (f) Mean annual temperature (from 1979 to 2013). (g) Soil pH (from 1960 to 2020).

We found distinct distribution patterns among the proportion of spinescent mimosoid species, large herbivore richness, and environmental factors across global assemblages (Fig. 1). Globally, the highest proportions of spinescent mimosoids (Fig. 1a) occur in seasonally dry tropical vegetation of the succulent biomes, including north‐east Brazil (Caatinga), southwest Madagascar, parts of Mexico, the Horn of Africa, the Karoo Namib region, and the Gran Chaco. African savannas, particularly in the south and west (e.g. South Africa, Zimbabwe, Mozambique, and the sub‐Sahelian zone), also show high proportions of spinescence. By contrast, New World savannas such as the Cerrado show only modest proportions of spinescent species, comparable to levels in Amazonian rainforest and significantly lower than in African savannas. In the Americas, the highest spinescence proportions are tightly associated with the succulent biome. This contrasts with Africa, where high spinescence occurs in both succulent and savanna biomes. Meanwhile, the Amazon, Congo basin, and most of Australia show low spinescence proportions. African savannas show exceptionally high extant herbivore richness (Fig. 1b), whereas northern Mexico, southern Brazil, Argentina, and Paraguay show the highest extinct herbivore richness (Fig. 1c). Globally, mean burn area coincides with savanna distribution (Fig. 1d), while dry‐season length (Fig. 1e) is highest in the succulent biome and Australian and southern African savannas. Mean annual temperature (Fig. 1f) is highest in tropical and subtropical regions, including the Amazon Basin, equatorial Africa, and northern Australia, whereas cooler conditions prevail at higher latitudes. Soil pH (Fig. 1g) is associated with dry season length and varies strongly across biomes, with relatively alkaline soils in arid and semi‐arid regions of the succulent biome in south‐west and north‐east Africa, parts of Mexico, and north‐east Brazil, as well as large parts of Australia, and more acidic, less fertile soils in humid tropical forests such as the Amazon and Congo Basin and the savannas of the Cerrado.

### Extant and extinct herbivores determine the global distribution of the proportion of spinescent species

**Fig. 2:**
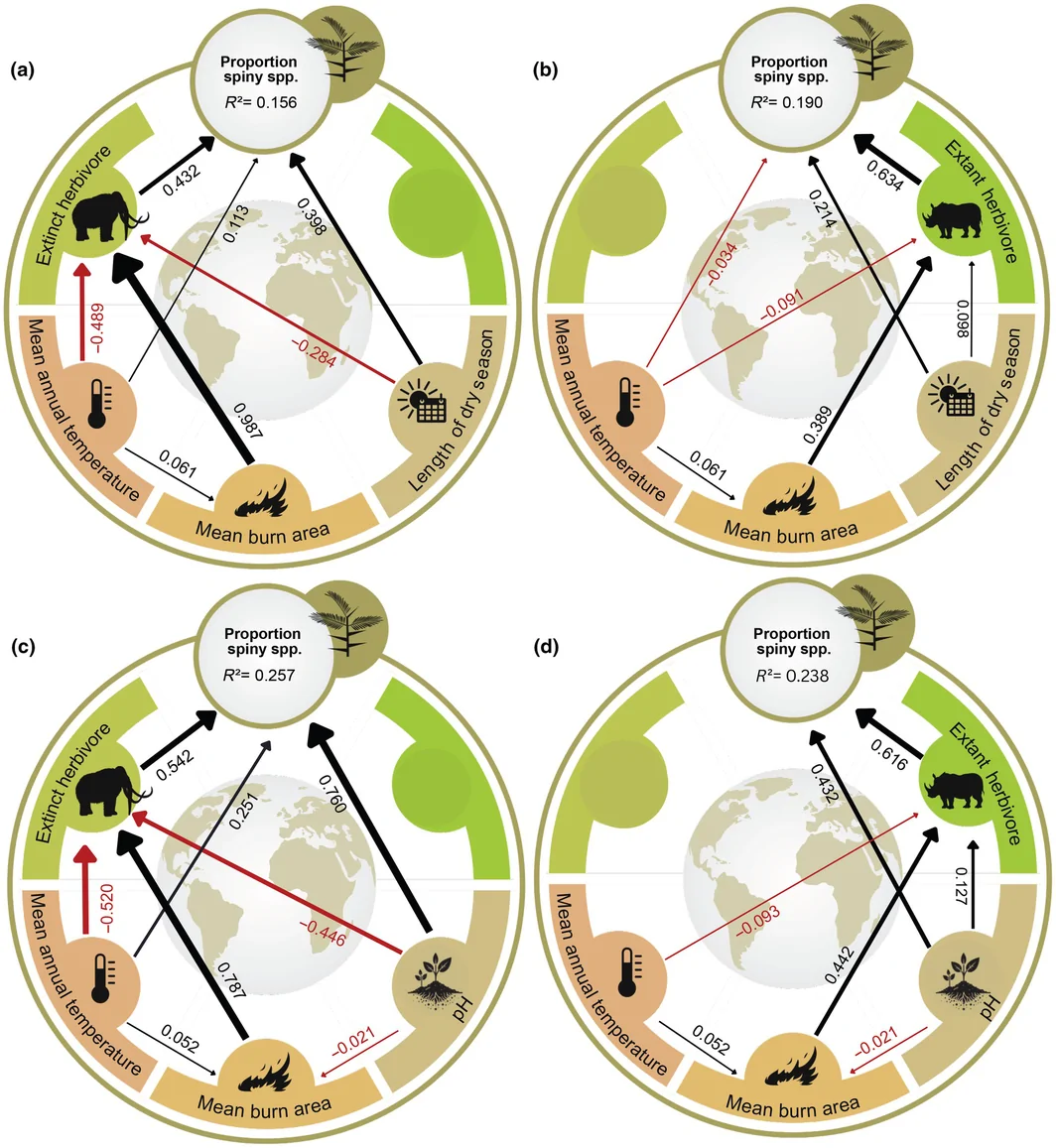
Global determinants of the distribution of proportion of spiny mimosoid species across assemblages. Structural equation models (SEMs) showing the direct and indirect effects of extant and extinct large herbivore species richness and environmental factors on the proportion of spiny (axillary spine, prickle, spinescent shoot, or stipular spine) mimosoid species across assemblages with a minimum of 3 mimosoid species. Panels (a–d) show global models (*n* = 3146 grid cells) with (a) extinct herbivore scenario and length of dry season, (b) extant herbivore scenario and length of dry season, (c) extinct herbivore scenario and soil pH, and (d) extant herbivore scenario and soil pH. Variable description and units are provided in (Table 1). Boxes represent variables, and arrows indicate the direction of the effects. Arrow thickness is proportional to the strength of the relationships (i.e. standardized path coefficient), with black and red arrows representing significant (*P* < 0.05) positive and negative path coefficients, respectively. The *R* ^2^ value reflects the total variation explained in the response variable. SEMs across assemblages with a minimum of 1 and 5 mimosoid species can be found in Supporting Information Tables S6 and S7a–d.

Global SEMs showed that soil pH explained more variation in spiny species proportion than dry‐season length, in both the extant and extinct herbivore richness scenarios (*R* ^2^: extinct soil pH = 0.257; extinct dry‐season = 0.156; extant soil pH = 0.238; extant dry‐season = 0.190; Fig. 2a–d; Table S3a–d). Furthermore, the models including extinct herbivore richness explained more variation than the models including extant herbivore richness.

In dry‐season length models, herbivore richness (extant or extinct) was the strongest predictor of spiny species proportion, whereas dry‐season length showed a smaller direct effect, and a minor effect of temperature was detected. For both extant and extinct herbivore scenarios, herbivore richness was higher in cooler areas of the (sub)tropics with high incidence of fire. However, the length of the dry season showed contrasting effects with herbivore scenarios: it negatively influenced extinct herbivore richness but had a positive effect on extant herbivore richness (Fig. 2a,b; Table S3a,b).

In pH‐based models, soil pH exerted the strongest direct effect on the proportion of spiny species, followed by a strong positive effect of extinct herbivore richness and a weaker effect of extant herbivore richness. Temperature had a positive direct effect on spiny species in the extinct herbivore model but none in the extant herbivore model. Similar to the dry‐season model, herbivore richness (extinct or extant) was higher in cooler areas of the (sub)tropics with high incidence of fire. Furthermore, soil pH showed contrasting effects with extant and extinct herbivore scenarios: extinct herbivore richness increased on more acidic soils, whereas extant herbivore richness increased on more alkaline soils. Areas with high extant and extinct herbivore richness also experienced higher fire frequency, and fires were more frequent in warmer regions with higher soil pH (Fig. 2c,d; Table S3c,d).

### Continental variation in determinants of the proportion of spiny species

**Fig. 3:**
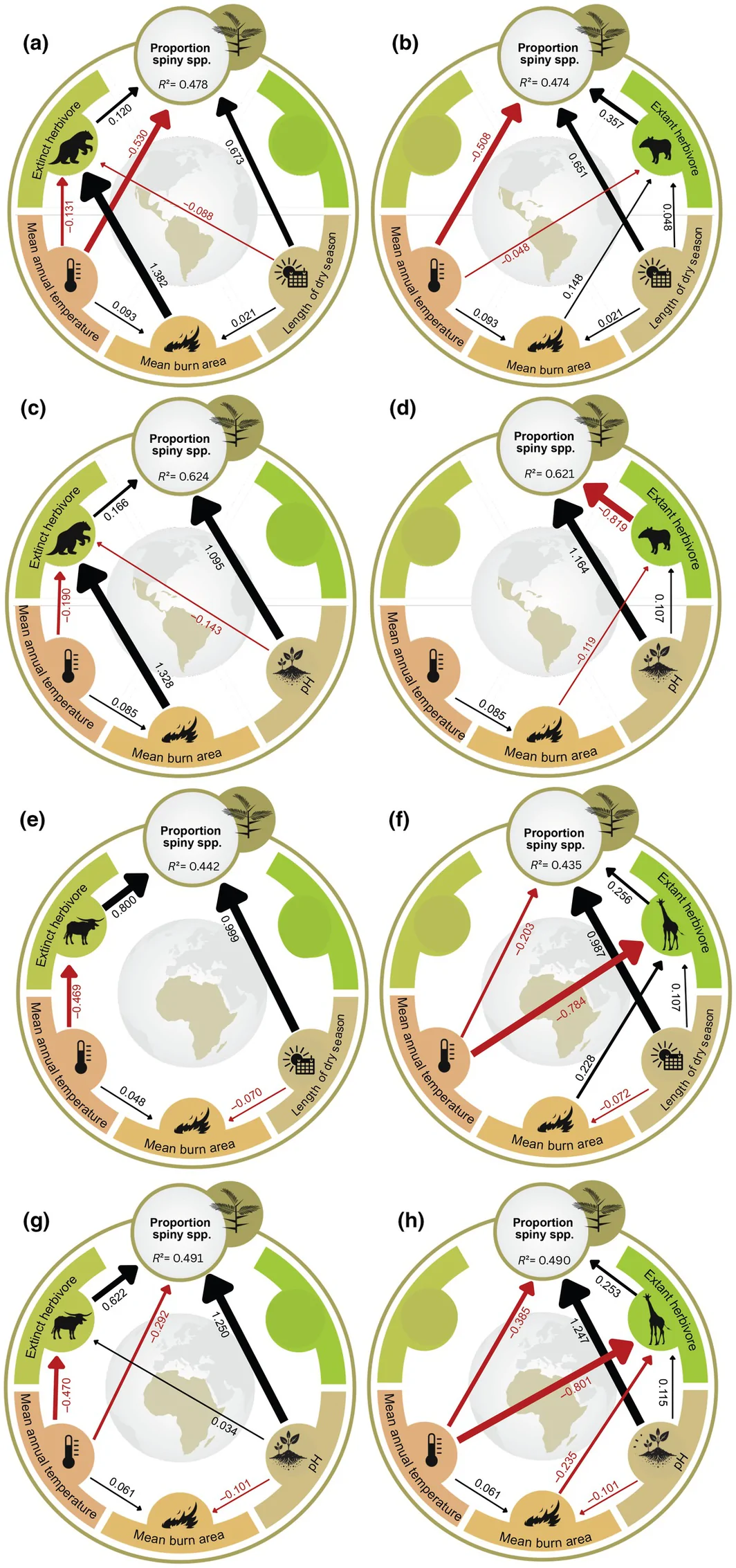
Continental determinants of the distribution of proportion of spiny mimosoid species across assemblages. Panels (a–d) show models for the Americas (*n* = 1378 grid cells) and panels (e–h) show models for mainland Africa (*n* = 643 grid cells). Within each region, panels are arranged as paired models that differ in herbivore type (extant vs extinct) and environmental predictor (dry‐season length vs soil pH). Boxes represent variables, and arrows indicate the direction of the effect. Arrow thickness is proportional to the strength of the relationship (i.e. standardized path coefficient), with black and red arrows representing significant (*P* < 0.05) positive and negative path coefficients, respectively. The *R* ^2^ value reflects the total variation explained in the response variable.

Across continents, climate and soil conditions were the primary determinants of spiny species proportion, with herbivore richness acting as a secondary, context‐dependent driver (Fig. 3). Furthermore, more variation in the proportion of spiny species was explained at continental than global scales (*R* ^2^ global = 0.156–0.257; *R* ^2^ continent = 0.435–0.624), with similar explanatory power of extant and extinct herbivore richness. Additionally, soil pH models explained more variation in spinescence than dry‐season models in the Americas, but not in Africa.

#### Americas

In dry‐season models, dry‐season length was the strongest predictor of the proportion of spiny species, followed by mean annual temperature and herbivore richness (Fig. 3a,b; Table S4a,b). The effect of extant herbivore richness was substantially higher than the effect of extinct herbivore richness. Furthermore, spiny species were more common in cooler areas, contrasting with the global model where spinescence was associated with warmer areas. Herbivore richness was also higher in cooler areas and in areas with frequent fires, in both extant and extinct herbivore scenarios. However, similar to the global model, extant herbivore richness increased with longer dry seasons, whereas extinct herbivore richness was higher in regions with shorter dry seasons. Mean burn area increased with both temperature and dry‐season length.

In pH‐based models, soil pH was the strongest direct predictor of spiny species proportion under both herbivore scenarios, followed by herbivore richness, and no effect of temperature on spinescence was detected (Fig. 3c,d; Table S4c,d). Interestingly, extinct herbivore richness had a positive direct effect on spiny species proportion, whereas extant herbivore richness had a negative effect. Similar to the global model, extinct herbivore richness increased on more acidic soils, whereas extant herbivore richness increased on more alkaline soils. Only extinct herbivore richness increased in cooler areas with more fire, whereas mean burn area was lower in areas with higher extant herbivore richness. Mean burn area increased with temperature only.

#### Africa

In dry‐season models, dry season length was the strongest predictor of spiny species proportion, followed by herbivore richness, and with stronger effects of extinct than extant herbivore richness (Fig. 3e,f; Table S5a,b). Spiny species proportion was higher in cooler areas under the extant herbivore scenario. Herbivore richness (extant or extinct) increased in cooler areas, and extant herbivore richness increased in regions with longer dry seasons and more fire. Mean burn area declined with increasing dry‐season length and under lower temperatures.

In pH‐based models, soil pH was also the strongest predictor of spiny species proportion, followed by herbivore richness and lower temperatures, with stronger effects of extinct than extant herbivore richness (Fig. 3g,h; Table S5c,d). Herbivore richness increased in more alkaline soils and under cooler temperatures, while extant herbivores were more frequent in areas with lower fire frequency. Mean burn area increased under lower soil pH and under higher temperatures.

### Spatial autocorrelation and randomizations

Raw spinescence and nonspatial model residuals showed strong positive spatial autocorrelation (global Moran's *I* = 0.55–0.62, *P* < 0.001; Fig. S2). SAR models indicated that the direct effects of extant herbivore richness and temperature on spiny species proportion were no longer significant (Table S3a–d), reducing confidence in the strength and independence of these associations.

Standardized coefficients from randomized spinescence across species indicated that observed effects for extant and extinct herbivore richness on spinescence from the empirical models lay outside the central 95% of null distributions from 1000 randomizations. Effects of dry‐season length, mean annual temperature, and soil pH also exceeded null expectations (Fig. S3).

### Evolution of spinescence through geological time

**Fig. 4:**
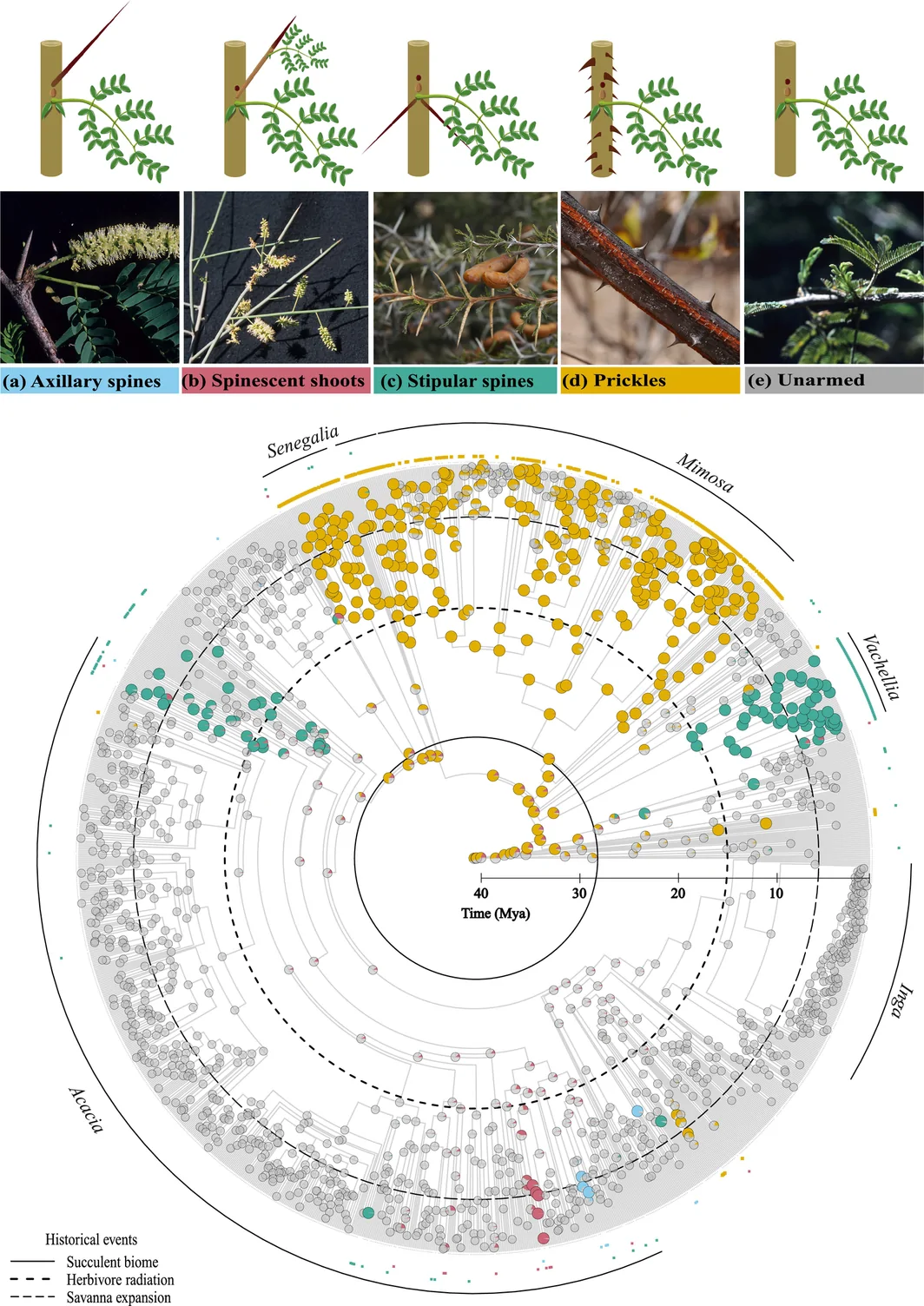
Evolution of spinescence in mimosoid legumes. Pie charts on nodes represent the probability of the four different spinescence types and absence of spinescence (‘unarmed’) under the best fitting models (i.e. All Rates Different, ARD; Supporting Information Table S8). Prominent mimosoid genera are highlighted at the tips of the phylogenetic tree. Biotic and environmental shifts are indicated with symbols and rings placed at their period of occurrence, with probable establishment of the succulent biome at *c*. 34–23 million years ago (Ma; Gagnon *et al*., 2019), radiations of large mammalian herbivores (e.g. bovids) in the mid‐Miocene at *c*. 15 Ma (Charles‐Dominique *et al*., 2016), and expansion of savannas starting at *c*. 8–3 Ma (Edwards *et al*., 2010). Coloured squares around the perimeter indicate observed tip states for extant species; species with multiple spinescence types are represented in multiple radial rows. Representative taxa illustrating spinescence types are shown above the tree: (a) *Neltuma juliflora* (axillary spines), (b) *Neltuma kuntzei* (spinescent shoots) (c) *Strombocarpa ferox* (stipular spines), (d) *Mimosa ophthalmocentra* (prickles), and (e) *Calliandra tumbeziana* (unarmed) (all photos by Colin Hughes). Botanical illustrations (a–e) were prepared by Ade Prasetyo Agung.

The ARD model best fitted the evolution of each spinescence type (Table S8). Ancestral state reconstructions show that spinescence evolved convergently with multiple independent origins across mimosoids' *c*. 40 Myr evolutionary history and reveal strong phylogenetic structuring of spinescence types (Fig. 4). Prickles are concentrated within *Mimosa*, whereas stipular spines are prominent in *Vachellia*. Other spinescence types (spinescent shoots, axillary spines) occur less frequently, and many internal nodes are reconstructed as unarmed.

Temporal mapping of reconstructed states suggests that some transitions to spinescence coincide with major ecological change (Fig. 4). Stipular spines show the earliest well‐supported origin dating to *c*. 33.3 million years ago (Ma; 95% highest posterior density (HPD) interval: 33.2–33.3 Ma) near the Eocene–Oligocene boundary, prickles arose slightly later, at *c*. 29.8 Ma (95% HPD: 29.7–30.0 Ma). These early origins coincided with the emergence of the succulent biome *c*. 34–23 Ma (Gagnon *et al*., 2019). However, the main diversification of spinescent lineages occurred later, broadly coinciding with the expansion of savannas during the mid–late Miocene (*c*. 8–3 Ma) (Edwards *et al*., 2010) and bovid diversification spikes (*c*. 15 Ma) (Charles‐Dominique *et al*., 2016). Axillary spines first appeared *c*. 16.1 Ma (95% HPD: 16.0–16.7 Ma), while spinescent shoots appeared *c*. 15.6 Ma (95% HPD: 14.3–17.9 Ma). Several axillary spine species belong to clades (e.g. *Neltuma*, *Chloroleucon*) that are represented by relatively few sampled tips, reducing the phylogenetic signal available for ancestral state estimation.

## Discussion

We used SEMs to disentangle the direct and indirect effects of herbivory, climate, soil, and fire on global and continental patterns of spinescence (Fig. 1). Using mimosoids as a model clade, our findings support the hypothesis (H1) that extant and extinct large herbivores are key predictors of the prevalence of spinescent species across global assemblages, although their relative influence differs among continents, and ‘present natural’ (extinct) scenarios perform better than extant only scenarios at global scale (Figs 2, 3). We also found support for the hypothesis (H2) that dry‐season length, soil pH, temperature and fire shaped spiny species proportion both directly and indirectly through their effects on herbivore richness (Figs 2, 3). Our SEMs highlight the complexity of the interactions between herbivores and environmental factors in structuring spinescence. Finally, we show that spinescence evolved multiple times independently across mimosoid lineages (Fig. 4), consistent with the repeated and convergent evolution of defence strategies.

### Herbivores influence the broad‐scale distribution of spinescence

Across SEMs including dry‐season length (Figs 2a,b, 3a,b,e,f) and soil pH (Figs 2c,d, 3c,d,g,h), the positive relationship between mammalian herbivore richness and spinescence was prominent and consistently detected, but effect strengths differed among continents and model formulations. In the global dry‐season model, herbivore richness (extant or extinct scenario) was the strongest direct predictor of spiny species proportion, whereas in the global pH model, soil pH was the strongest predictor, and herbivory was secondary. This persistent herbivory effect on spinescence is consistent with the evolutionary arms‐race framework (Dawkins & Krebs, 1979) in which sustained browsing pressure selects for structural defences. Where herbivore richness is high and environmental conditions promote palatable foliage, plants experience repeated tissue loss concentrated on young, nutrient‐rich shoots, increasing the fitness benefits of traits that reduce bite size, slow intake rates, and deter browsing. Spinescence reduces bite size and feeding efficiency and increases handling time of medium‐to‐large browsers, thereby lowering tissue loss and increasing plant fitness under heavy herbivory (Cooper & Owen‐Smith, 1986; Wilson & Kerley, 2003).

It remains unclear to what extent spinescence is an effective defence against the largest herbivores (but see (Goheen & Palmer, 2010)). In our dataset, very large megaherbivores (≥ 1000 kg) were rare (36 species), but we do show that the inclusion of extinct herbivores, many of which were over 1000 kg, consistently increased the amount of explained variation in the distribution of spinescence, and the effect was robust against spatial autocorrelation, indicating persistent ecological imprints of large extinct herbivores on modern spinescence patterns. For several mimosoids (e.g. *Mimosa tricephala*, *Sphinga platyloba*, and *Senegalia riparia*), recurved thorns have been hypothesized to deter large extinct browsers, including ground sloths and gomphotheres (Janzen & Martin, 1982); however, such examples remain speculative. Testing whether spinescence values (e.g. size, sharpness or inducibility) vary with present and extinct megaherbivores could further elucidate herbivore–mimosoid interaction patterns, with spinescence traits likely varying among herbivore lineages and feeding strategies. Although spinescence might now face reduced selection, its persistence highlights the lingering impact of past herbivory. Similarly, megafaunal fruits in Neotropical palm species may be relicts of dispersal by now extinct pre‐Quaternary megafauna (Lim *et al*., 2020; Wölke *et al*., 2023).

Interestingly, the relative effect of extinct vs extant herbivores was stronger in Africa than in the Americas, contrasting our expectation. This finding is nonetheless consistent with research on African savannas, where spines were strongly associated with evolutionary legacies of past herbivore regimes (Charles‐Dominique *et al*., 2016; Gélin *et al*., 2023). In the Americas, the effect of extinct herbivores remains positive, supporting the legacy effect in the Americas (Fig. 3c). By contrast, pH appears to counteract or replace the effect of extant herbivores, as indicated by a strong negative association between extant herbivore richness and the proportion of spinescent mimosoids (Fig. 3d). This aligns with Neotropical evidence that megafaunal history and soil fertility jointly structure broad‐scale patterns in physical defences (Dantas & Pausas, 2022). Nonetheless, the region's diverse extinct megafauna implies historically intense browsing pressure on woody plants, potentially favouring mechanical defences (Owen‐Smith, 2013).

Although distributions of extinct large herbivores are supported by fossil evidence, museum specimens and the literature (Faurby *et al*., 2018), their geographic scope should be interpreted with caution because reconstructed ranges remain vulnerable to spatial gaps from sampling biases and uneven fossil preservation (Holland, 2016). The consistency of effects across SEMs nonetheless suggests that these uncertainties do not obscure the broader signal of megafaunal legacy on spinescence.

### Environmental modulators of herbivory effects on spinescence

Environmental conditions, especially soil pH and dry season length, had both direct and indirect effects on global spinescence patterns, by affecting extant and/or extinct herbivore richness. Higher soil pH and longer dry seasons were consistently associated with increased extant herbivore richness and a greater prevalence of spinescent species. This confirms our expectation that herbivore pressure may increase on more fertile, less acidic soils where nutrient availability enhances leaf quality and attraction to herbivores (Bell, 1982; Tomlinson *et al*., 2016; Dantas & Pausas, 2022), and in seasonally dry systems, in which brief but synchronized flushes of palatable leaves at the onset of rainy seasons may intensify browsing and favour defences (Silva *et al*., 2020). This is consistent with continental‐scale analyses showing that pH is the strongest predictor of stem spines in Neotropical woody floras (Dantas & Pausas, 2022), and that higher soil fertility supports higher large‐mammal densities in African savannas, thus increasing pressure for plant defence (Wigley *et al*., 2015, 2018; Dantas & Pausas, 2022). Additionally, Pliocene aridification promoted the expansion of open grasslands that supported higher carrying capacities for large grazers and browsers (Bobe, 2006), illustrating how drought influences herbivore richness through vegetation structure and productivity rather than via direct physiological constraints.

Because soil pH and length of dry season were highly correlated across the distribution of mimosoids, we could not disentangle their relative effects within a single SEM framework. Most mimosoid species are nitrogen‐fixers and many are adapted to dry areas with alkaline soils (Steidinger *et al*., 2019; Dantas & Pausas, 2022). Furthermore, nutrient availability is generally less constraining in dry conditions, because nutrients can surface in dry soils, making them more accessible to plants (Hartemink & Barrow, 2023). At continental scales, soil pH is highly negatively correlated with climate aridity (Tomlinson *et al*., 2025); this suggests that the proportional abundance of spiny Mimosoideae may be more strongly determined by the relative change in soil nutrient supply to water supply (Tomlinson *et al*., 2016) than by limitation on plant productivity (dry season length). This may explain why models including soil pH consistently explained more variation than those including length of the dry season.

Globally, fire had an indirect effect on spinescence through its positive association with herbivore richness. This likely reflects a shared association with open ecosystems, in which both fire prevalence and herbivore richness increase. At continental scales, its effect differed: in the Americas, fire positively affected both extant and extinct herbivore richness in dry‐season models, whereas in pH‐based models it was negatively associated with extant herbivore richness but positively associated with extinct herbivore richness. In Africa, fire positively affected extant herbivore richness but not extinct herbivore richness in dry‐season models, while in pH‐based models it was negatively associated with extant herbivore richness and showed no association with extinct herbivore richness. This is consistent with the legacy of megafaunal collapse in the Americas, thereby reducing biomass consumption and trampling, and increasing fuel loads and fire activity (Galetti, 2004; Bond & Keeley, 2005). In Africa, the balance between fire and herbivores depends on nutrient and water availability: fire tends to dominate in high‐rainfall, nutrient‐poor systems, whereas herbivores dominate in drier, nutrient‐rich systems (Archibald & Hempson, 2016). Previous studies have shown that fire–browser interactions can be bidirectional and dependent on vegetation structure, fire intensity, and resource availability (van Langevelde *et al*., 2003; Archibald & Hempson, 2016).

In the continental models, herbivore effects were consistently weaker than environmental effects on the proportion of spiny species, indicating scale‐dependent drivers of spinescence. Temperature had a strong direct and/or indirect effect on spinescence at continental scales, with the proportion of spines and herbivore richness increasing under relatively cooler climates. Importantly, this pattern reflects cooler temperatures at subtropical latitudes rather than higher elevations as mimosoids are essentially absent from montane areas (> 2500 m elevation) such as the Andes. Lower temperatures reduce primary productivity, increasing the cost of biomass loss to herbivory and favouring greater allocation to defence (Tomlinson *et al*., 2025).

### The absence of spines, thorns, and prickles in certain regions

Our findings reveal low proportions of species bearing prickles, axillary spines, spinescent shoots, or stipular spines in certain regions and biomes (Figs 1, S4). For example, in rainforests, plants often rely on chemical defenses to deter mainly insect herbivores (Coley *et al*., 1985; Kursar *et al*., 2009; Mithöfer & Boland, 2012). In the Amazonian mimosoid genus *Inga*, co‐occurring species show greater divergence in chemical defenses than expected by chance, consistent with herbivore‐driven selection (Schley *et al*., 2025). Spinescence is also notably less prevalent in mimosoid lineages in Australia than in Africa or South America. Pliocene increases in lineages bearing spinescence in Australia have been reported (Gélin *et al*., 2023; Nge *et al*., 2023), but these radiations involved relatively few species. This pattern in spinescence parallels the continent's relative scarcity of succulents (Holtum *et al*., 2016; Nge *et al*., 2023) and deciduous species. Instead, there is a dominance of evergreen woody species with alternative spine morphologies, notably marginal and leaf‐tip spines (Tomlinson *et al*., 2025). Floristically, this pattern is reflected in the large (> 1000 species) mimosoid genus *Acacia*, which comprises mainly evergreen species with reduced phyllodinous leaves. In numerous cases, phyllodes are hardened and sharply pointed, potentially serving a defensive role (Tomlinson *et al*., 2025).

In the South American Cerrado, spinescent mimosoids are relatively scarce and resemble Amazonian levels (Fig. 1). Fire is a major driver of Cerrado vegetation openness (Durigan & Ratter, 2016), with soil properties also contributing (Goodland & Pollard, 1973). Given the exceptionally nutrient‐poor, acidic soils, intensive mammalian browsing pressure may have been limited, and selection may instead have favoured traits linked to fire persistence. Many woody plants in the Cerrado exhibit strong fire‐related strategies, including tough wood, corky bark, fire‐induced flowering, and resprouting, alongside chemical and mechanical traits such as latex, fruit spines, and sclerophylly, which may reflect multiple selective pressures, including both insect and vertebrate herbivory (Simon & Pennington, 2012; Dantas & Pausas, 2020, 2022). By contrast, Neotropical seasonally dry forests generally occur on less acidic, more fertile soils, with higher plant productivity and nutrient‐rich foliage, explaining the higher proportions of spinescent mimosoids in these areas.

### The evolution of spinescence

Although our spatial analyses show strong effects of herbivores on spinescence, ancestral state reconstructions reveal a more complex evolutionary history of spinescence types (Fig. 4). Spinescence evolved repeatedly and independently across the mimosoid phylogeny, suggesting recurrent exposure to similar selective pressures, while specific spine types likely reflect lineage‐specific developmental pathways.

Different spine types emerged at different times: stipular spines represent the earliest well‐supported origin, dating to *c*. 33.3 Ma (95% HPD: 33.2–33.3 Ma) near the Eocene–Oligocene boundary, while prickles arose slightly later, *c*. 29.8 Ma (95% HPD: 29.7–30.0 Ma). These origins broadly coincide with late‐Eocene (*c*. 39 Ma) spiny‐plant fossils from central Tibet and the emergence of open semi‐wooded vegetation (Zhang *et al*., 2022), shifts of several mimosoid lineages from tropical climates into seasonally dry succulent biomes (Gagnon *et al*., 2019; Ringelberg *et al*., 2023), and the (re‐)emergence of large herbivores after the ‘megaherbivore gap’ at *c*. 40 Ma (Smith *et al*., 2010; Onstein *et al*., 2022). This illustrates the complex abiotic–biotic context to the evolution of spinescence. Nevertheless, we observe ongoing rapid diversification and potentially ecological expansion of spinescent lineages through the mid‐ to late Miocene (from *c*. 15 Ma onwards, Fig. 4), similar to the diversification and expansion of C4 grasses and succulent lineages long after their origin (Edwards *et al*., 2010; Arakaki *et al*., 2011). By contrast, the emergence of axillary spines and spinescent shoots directly coincide with the spread of C4 grass‐dominated savannas and bovid diversification (Cerling *et al*., 1997; Beerling & Osborne, 2006; Edwards *et al*., 2010; Charles‐Dominique *et al*., 2016), suggesting renewed selection for structural defences in these open, herbivore‐rich landscapes.

The repeated emergence of spinescence in mimosoids highlights the complex interplay among ecological pressures, phylogenetic constraint, and resource allocation trade‐offs. Ancestral state reconstructions (Fig. 4) reveal strong phylogenetic structuring of spinescence (e.g. stipular spines are common in *Vachellia* and prickles in *Mimosa*). However, it remains unclear whether different spine types serve distinct ecological functions or primarily reflect structural origin. Patterns across species suggest possible associations between spinescence and growth form (Fig. S5), although these relationships remain to be explored in detail. Prickles are common in scrambling or climbing taxa, such as species of *Mimosa*, *Piptadenia* and *Senegalia*, but also occur in tree species, in the form of conical, sharp‐tipped prickles on tree trunks (e.g. *Cylicodiscus* and *Indopiptadenia*). By contrast, stipular and axillary spines are more frequent in shrubs and trees. Spinescent shoots are comparatively rare, possibly because they require modification of an entire shoot axis rather than smaller structures such as stipules or epidermal tissues. Defence allocation may also vary with plant life history strategy, with greater investment in structural defences in slow‐growing, longer‐lived species and reduced allocation in fast‐growing species (Armani *et al*., 2019). Accordingly, prickles and stipular spines may be favoured in productive environments that support rapid growth, whereas leaf spines may be associated with resource‐poor environments selecting for more conservative growth strategies (Campbell, 1986; Tomlinson *et al*., 2025). These observations raise the possibility that distinct spine types may also differ in function. Stipular and axillary spines may restrict access to leaves, whereas prickles may deter contact along shoots and, in climbing taxa, facilitate attachment to surrounding vegetation. However, spinescence function may vary within clades. In African *Senegalia*, nodal prickles may function like axillary spines, whereas smaller, more dispersed, inter‐nodal prickles may facilitate attachment in climbing species.

These observations highlight the potential for further investigation on the functionality, selective pressures and diversification of different defence strategies in mimosoids and other spinescent lineages. Trait‐ and climate‐dependent diversification models provide opportunities to test how historical shifts in drought and herbivore communities shaped defensive evolution in mimosoids and other spinescent angiosperms (e.g. Anest *et al*., 2024), while future work integrating specific spinescence function, growth form and plant architecture across biogeographical realms may clarify whether biome and environmental context influence the evolution of different spine types.

## Competing interests

None declared.

## Author contributions

REO and RSF designed the study, with input from JJR and CH. RSF collected the data and performed the analyses, with contributions from REO, JJR, CH, FJRW and EA. RSF and REO wrote the manuscript with contributions from JJR, CH, EA, FJRW and KWT.

## Disclaimer

The New Phytologist Foundation remains neutral with regard to jurisdictional claims in maps and in any institutional affiliations.

## Supporting information


**Fig. S1** Pearson's correlation matrix among variables used in the structural equation models.
**Fig. S2** Spatial distribution of spinescent mimosoid species and residuals from ordinary least‐squares and spatial error models.
**Fig. S3** Effects of extant and extinct herbivore richness, dry‐season length, soil pH and mean annual temperature on randomized global assemblages.
**Fig. S4** Global distribution of axillary spines, prickles, spinescent shoots and stipular spines in mimosoid legumes.
**Fig. S5** Association between plant growth form and spine type in Mimoseae.
**Notes S1** References used to score spinescence type for each taxon.
**Table S1** Glossary of morphological spinescence types in Mimoseae.
**Table S2** Extinct megaherbivore diet coverage by continent.
**Table S3** Standardized regression coefficients from the global structural equation model and spatial autoregressive model for assemblages containing at least three mimosoid species.
**Table S4** Standardized regression coefficients from the structural equation model and spatial autoregressive model for assemblages in the Americas containing at least three mimosoid species.
**Table S5** Standardized regression coefficients from the structural equation model and spatial autoregressive model for assemblages in Africa containing at least three mimosoid species.
**Table S6** Standardized regression coefficients from the global structural equation model for assemblages containing at least one mimosoid species.
**Table S7** Standardized regression coefficients from the global structural equation model for assemblages containing at least five mimosoid species.
**Table S8** Transition rates among armature types under equal‐rates and all‐rates‐different models.
Please note: Wiley is not responsible for the content or functionality of any Supporting Information supplied by the authors. Any queries (other than missing material) should be directed to the *New Phytologist* Central Office.

## Data Availability

All data needed to evaluate the conclusions in the paper are present in the paper and/or the Notes S1 and Tables S1–S8. All data and R scripts, including occurrence datasets and mammal and mimosoid trait datasets, are available from Zenodo and Github (doi: 10.5281/zenodo.19232275) (https://github.com/RachelSouzaFerreira/Mimosoid_Armature).
