# Supplementary material for: The complex interaction between mammalian herbivores, climate, soil and fire has shaped the evolution and distribution of plant spinescence across biogeographical realms
Source: New Phytol. 2026 Jul 22;251(6):3640–55. doi: 10.1111/nph.71376 (PMC13491257; doi:10.1111/nph.71376)
Supplement: Supplementary file 1 [file NPH-251-3640-s001.pdf]

New Phytologist Supporting Information

Article title: The complex interaction between mammalian herbivores, climate, soils and fire has shaped the evolution and distribution of plant spinescence across biogeographical realms

Authors: Rachel Souza Ferreira, Jens J. Ringelberg, Colin Hughes, Eduardo Arlé, Friederike J. R. Wölke, Kyle W. Tomlinson, Renske E. Onstein

Article acceptance date: 29 May 2026

Supplementary Figures

Figure S1: Pearson correlation matrix between variables used in SEMs. Abbreviations follow: Perc_spines = porportion of spinescent species, extant = extant herbivore richness, PN_total = extinct (present natural) herbivore richness, ph = soil pH, temp_chelsa = mean annual temperature, BA = mean burn area, lengthDry = number of dry months.

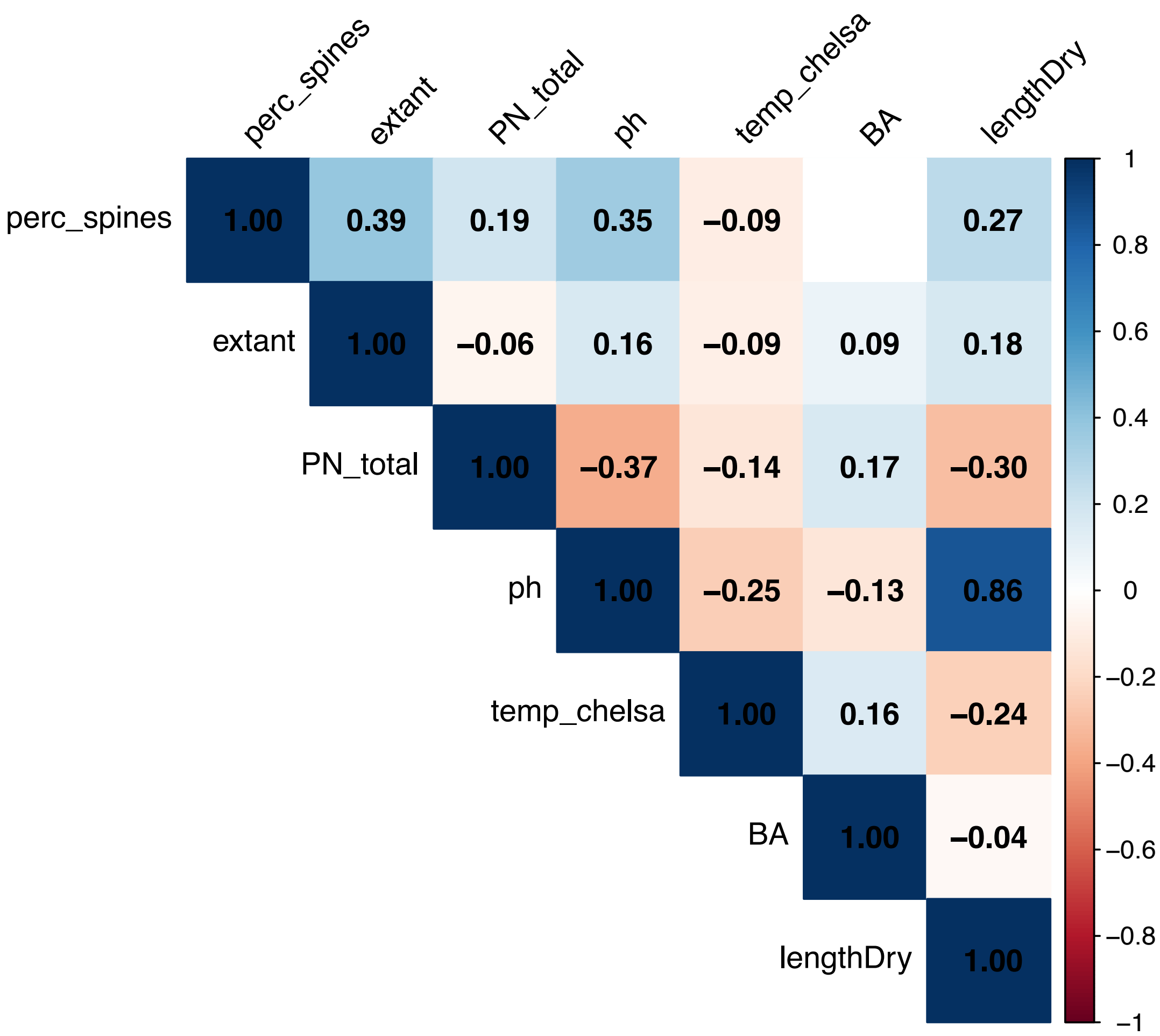

Figure S2: **Spatial autoregression models of spinescent mimosoid species and residuals from OLS and spatial error models**. Global maps of observed spinescence proportion and spatial patterns of model residuals for models including herbivore richness and environmental predictors. For each panel, the top map shows the observed proportion of spinescent mimosoid species per grid cell, followed by residuals from ordinary least squares (OLS) models and spatial autoregressive models (SAR). The OLS and SAR models evaluated the direct effects on spinescence proportion as detected in the structural equation models (SEM) but did not consider indirect effects. By comparing OLS and SAR models, we can evaluate whether direct effects as detected in OLS/SEM models are still supported after correcting for spatial autocorrelation in SAR models. Results of SAR models are provided in Table S3.

(a) Model including present-natural herbivore richness (extinct scenario) and dry season length.
(b) Model including extant herbivore richness and dry season length.
(c) Model including present-natural herbivore richness (extinct scenario) and soil pH.
(d) Model including extant herbivore richness and soil pH.

Values were linearly interpolated between grid-cell centroids for visualization. Spatial structure was modelled in the SAR framework, not within the structural equation models.

a) Extinct/dry season length

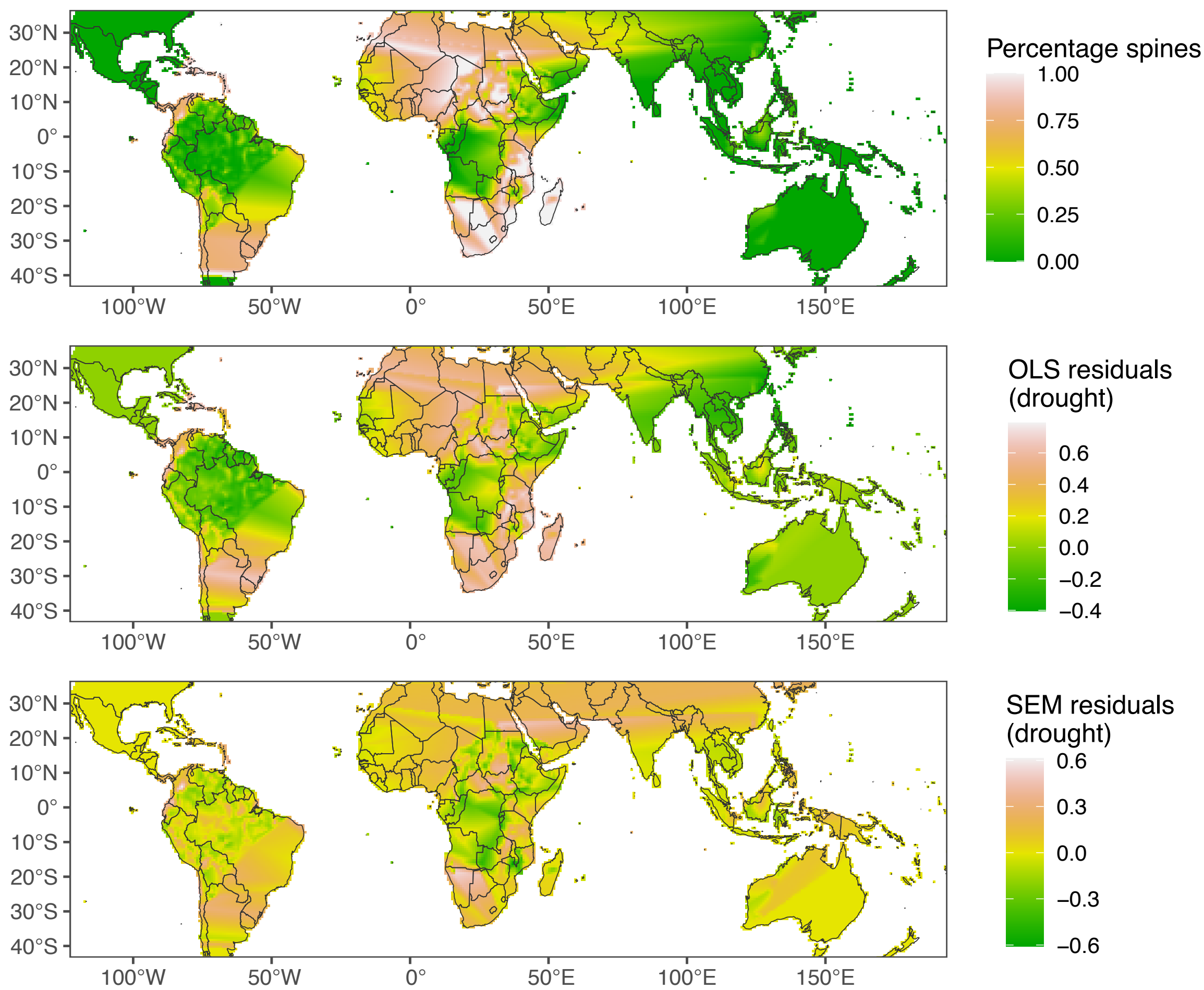

b) Extant/dry season length

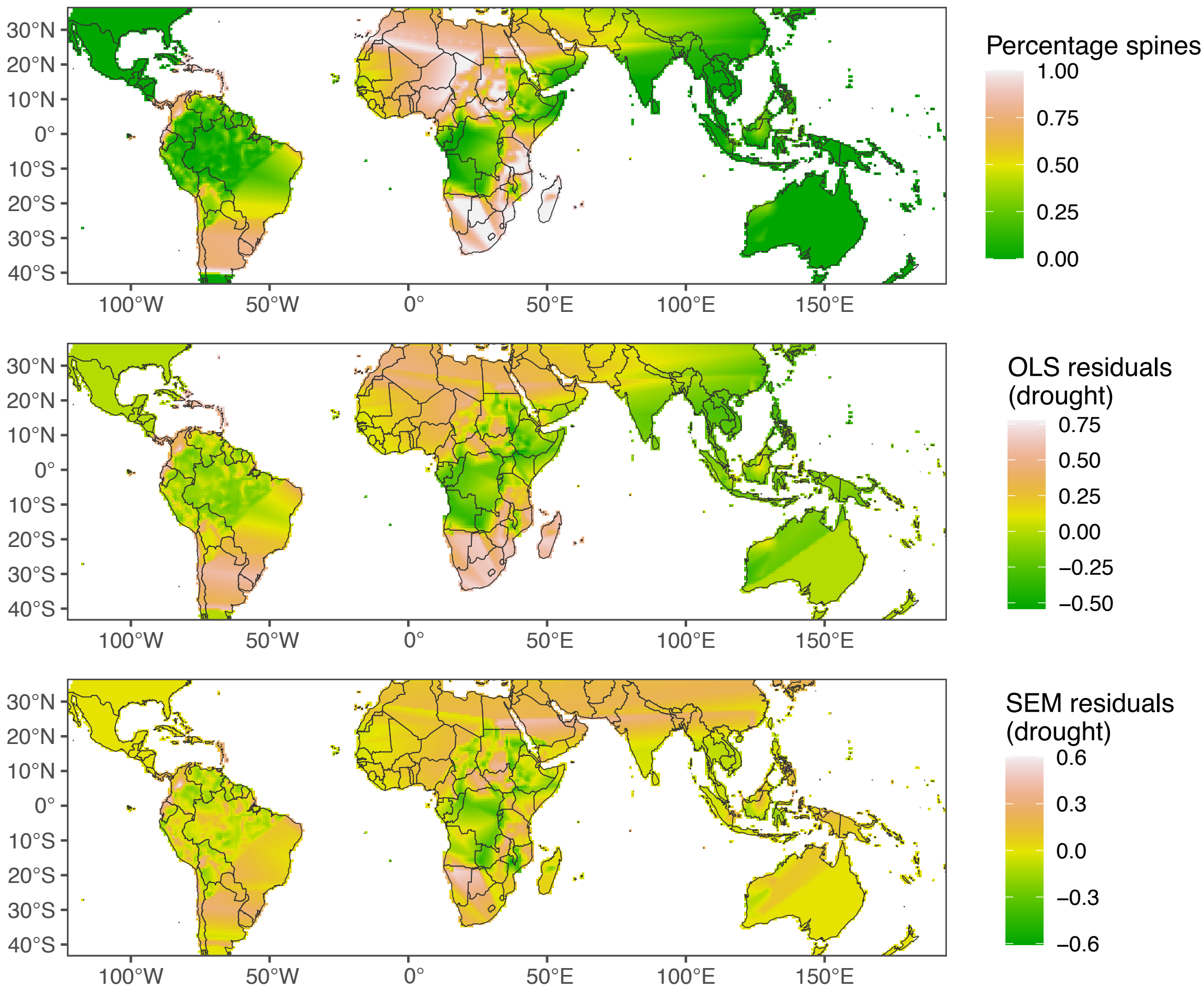

c) Extinct/pH

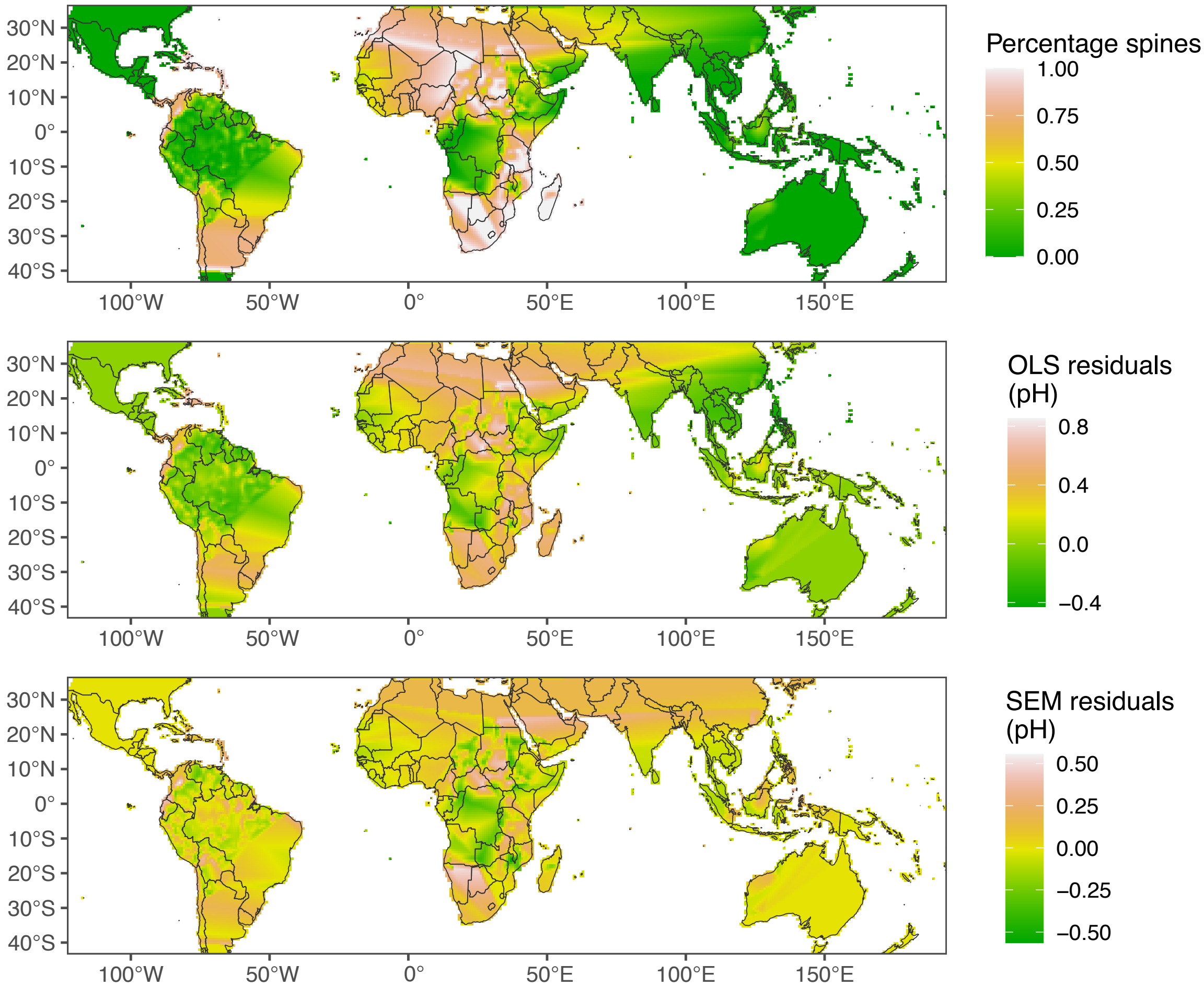

Percentage spines
1.00
0.75
0.50
0.25
0.00
OLS residuals (pH)
0.8
0.4
0.0
−0.4
SEM residuals (pH)
0.50
0.25
0.00
−0.25
−0.50
30°N
20°N
10°N
0°
10°S
20°S
30°S
40°S
100°W
50°W
0°
50°E
100°E
150°E

d) Extant/pH

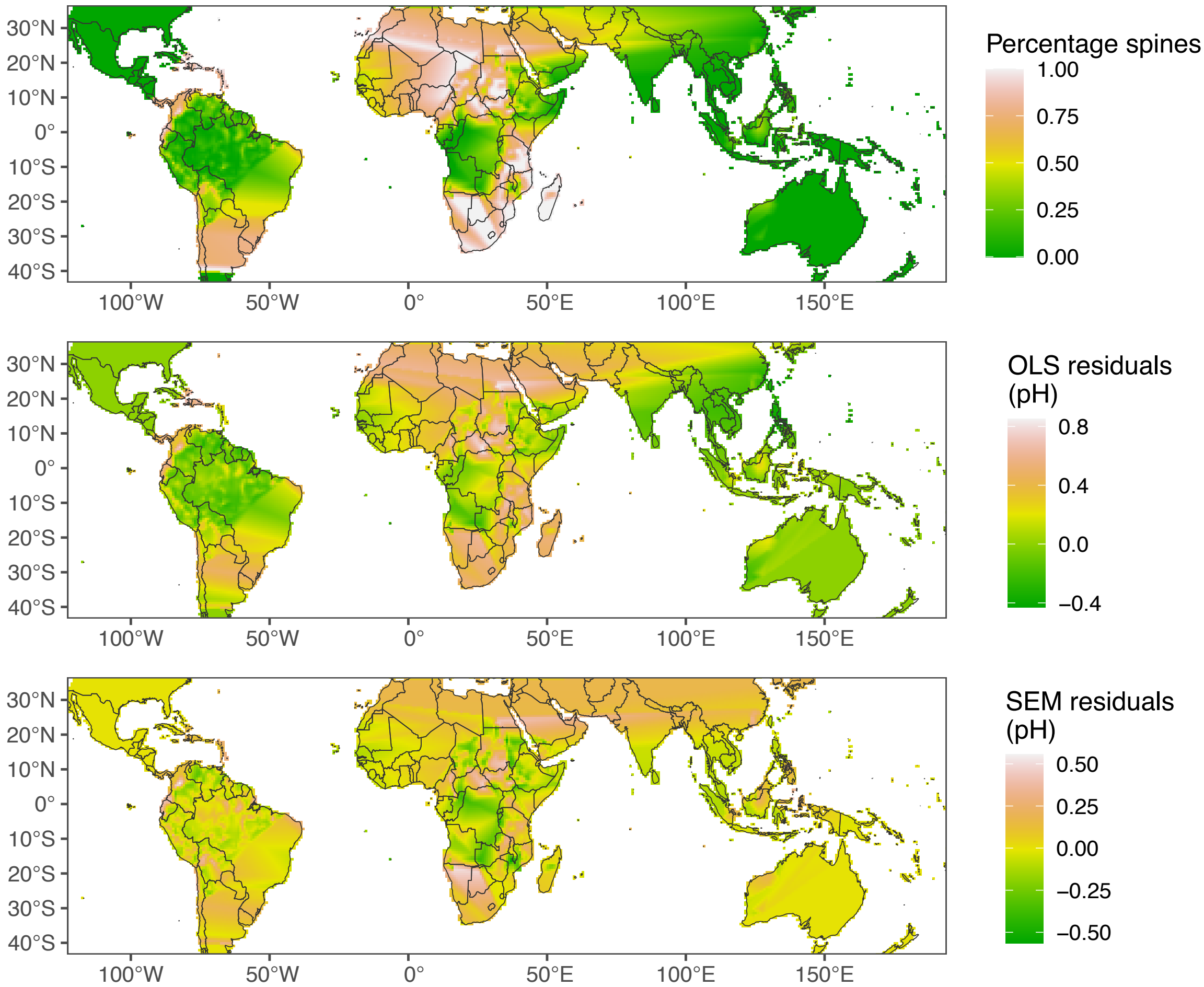
30°N
20°N
10°N
0°
10°S
20°S
30°S
40°S
100°W
50°W
0°
50°E
100°E
150°E
Percentage spines
1.00
0.75
0.50
0.25
0.00
OLS residuals (pH)
0.8
0.4
0.0
−0.4
SEM residuals (pH)
0.50
0.25
0.00
−0.25
−0.50

Figure S3: Effect of extant and extinct herbivore richness, number of dry months, soil pH and mean annual temperature on randomised global assemblages with spinescence. Grey bars represent the distribution of simulated standardized coefficients and red line represent the observed standardized coefficient from the SEMs.

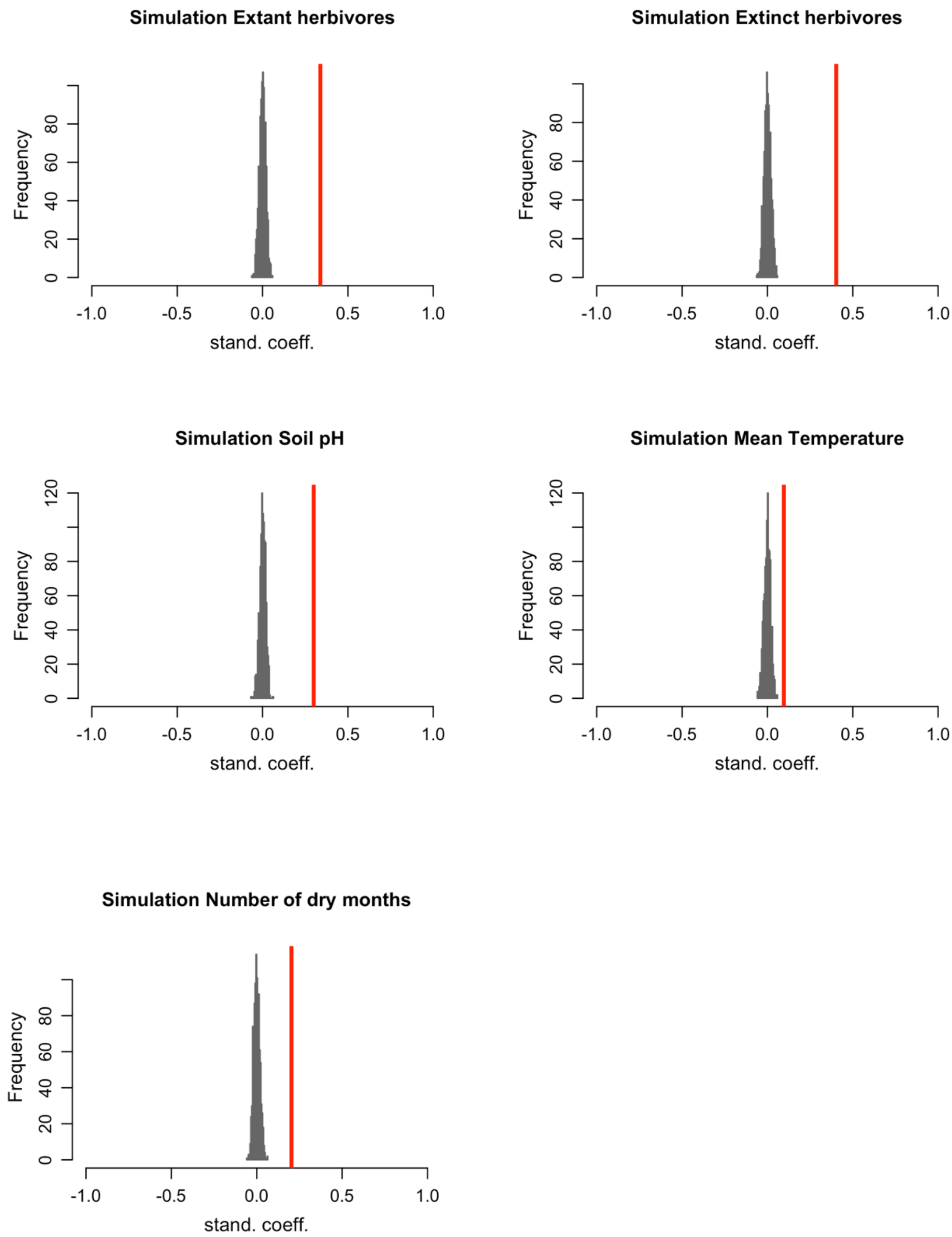

Figure S4: Global distribution of axillary spines, prickles, spinescent shoot and stipular spines in mimosoid legumes. Colours represent the number of spinescent species with per grid cell, grey indicates cells with no available spinescence data.

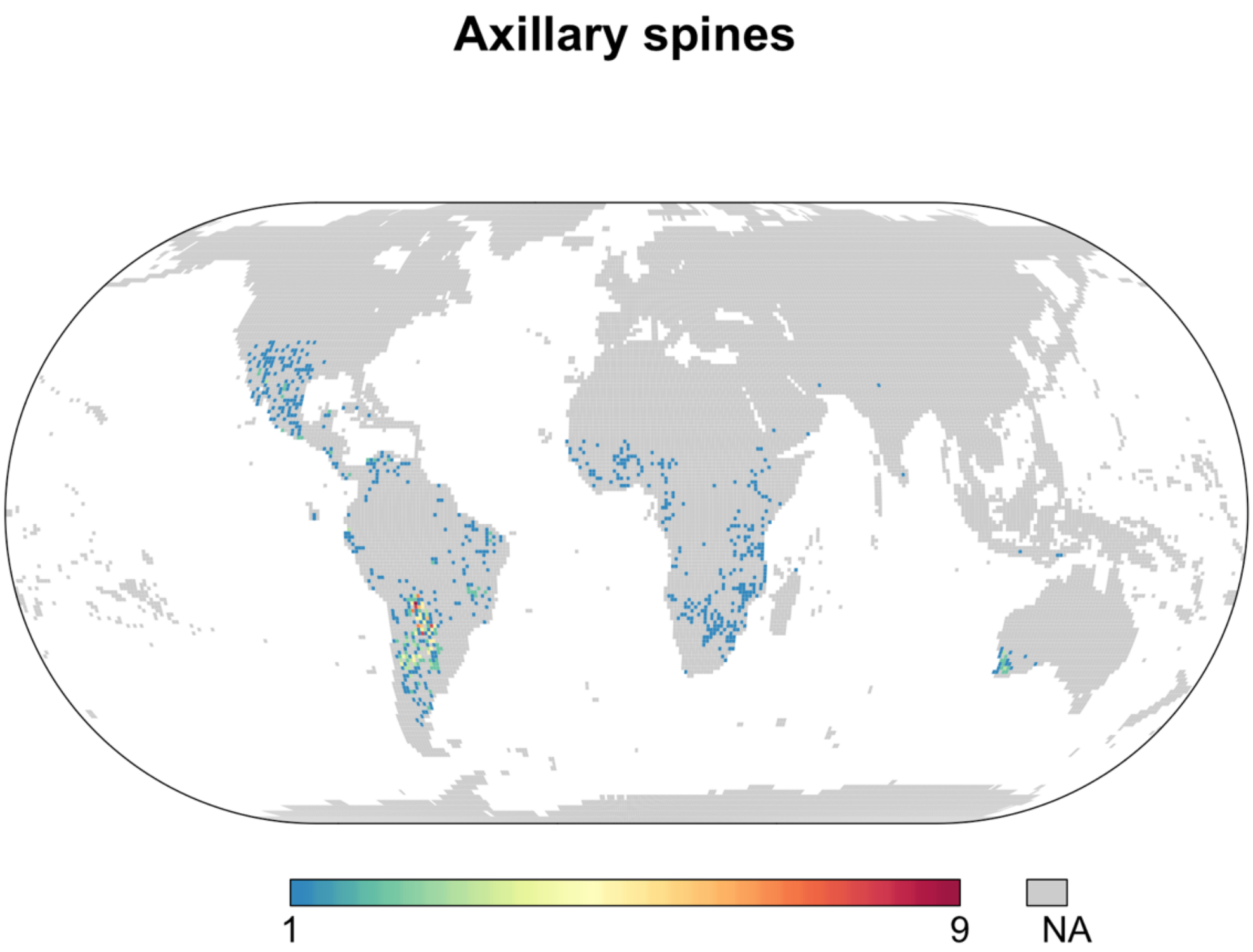

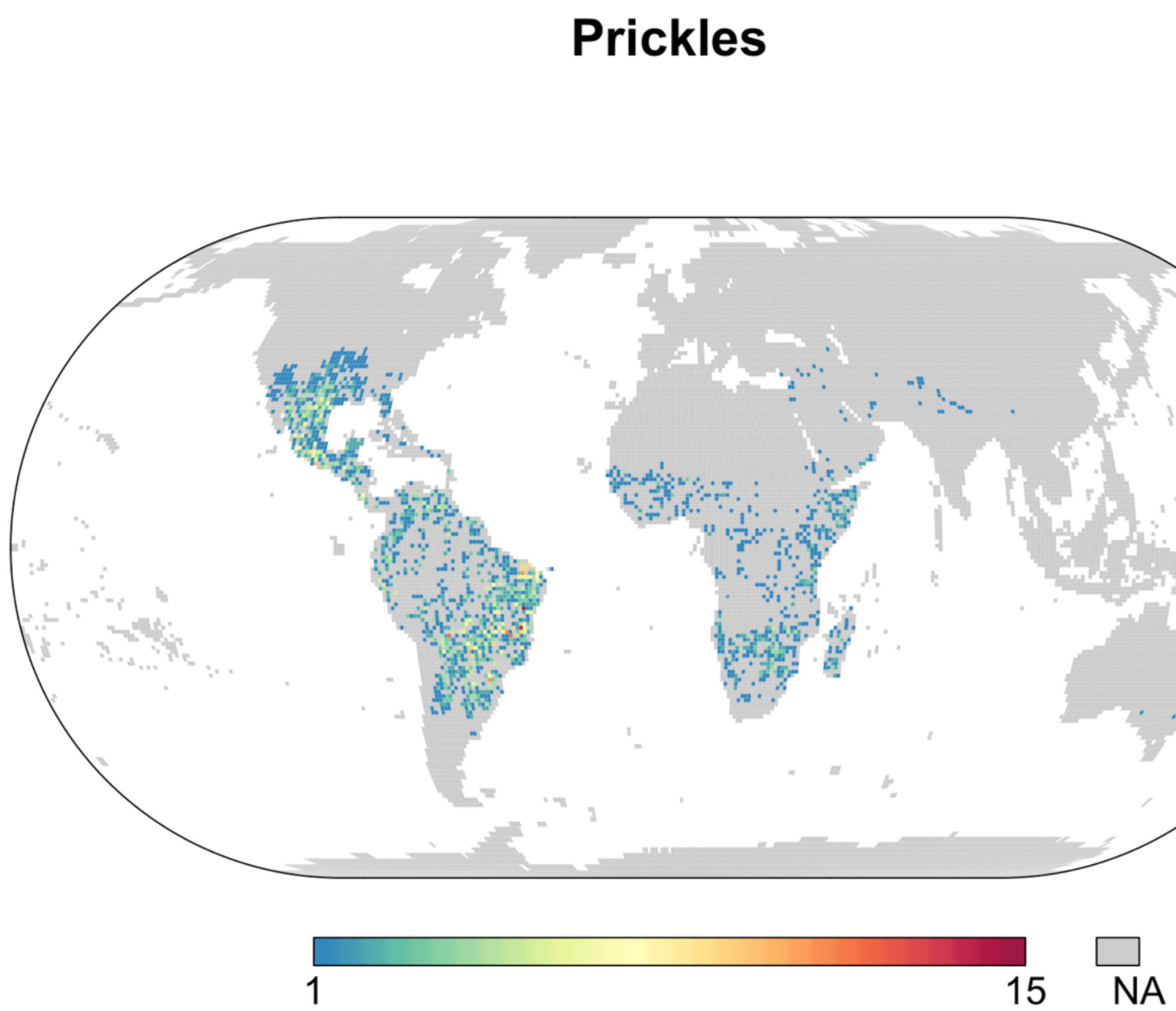

Prickles
1
15
NA

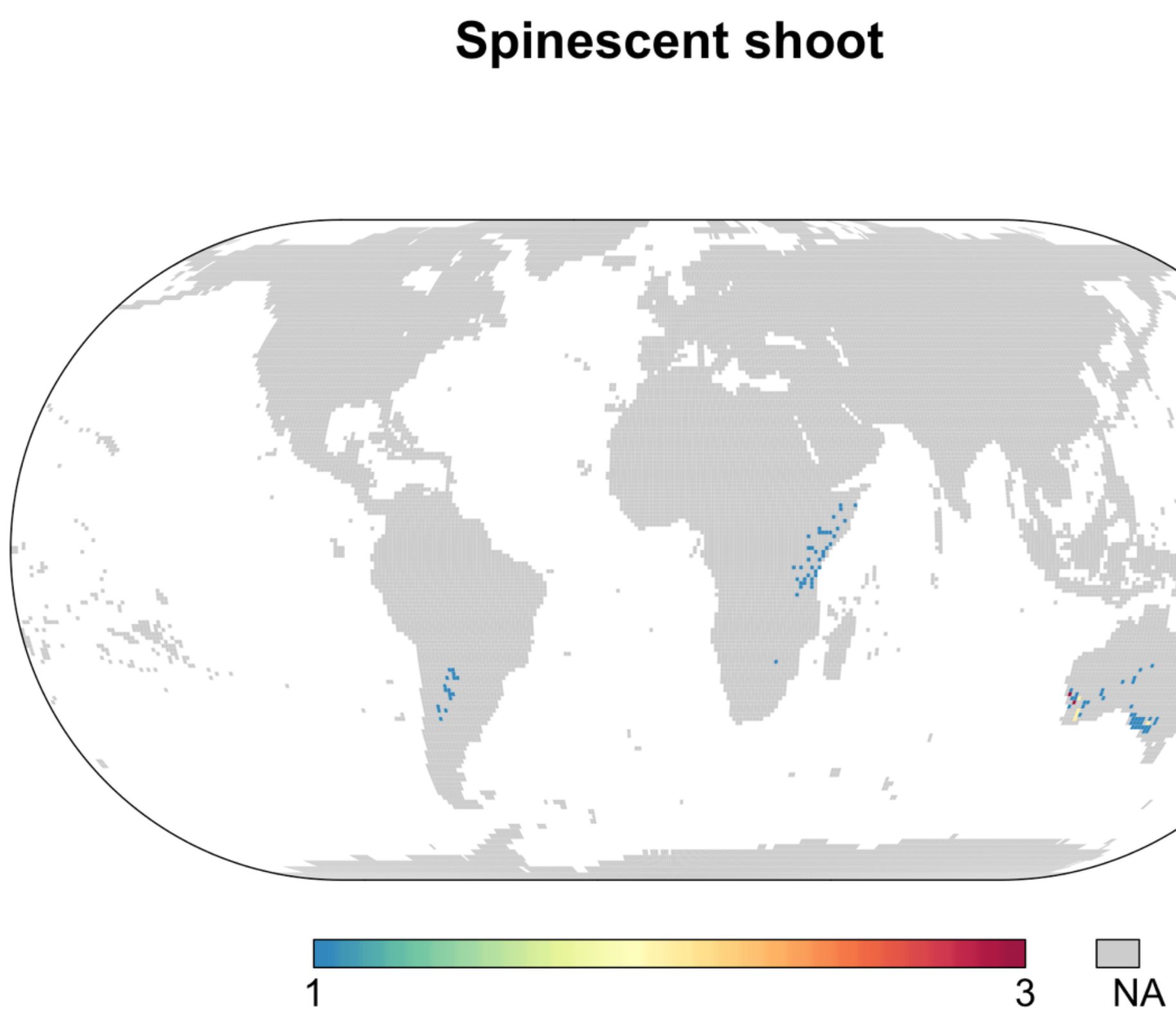

Spinescent shoot
1
3
NA

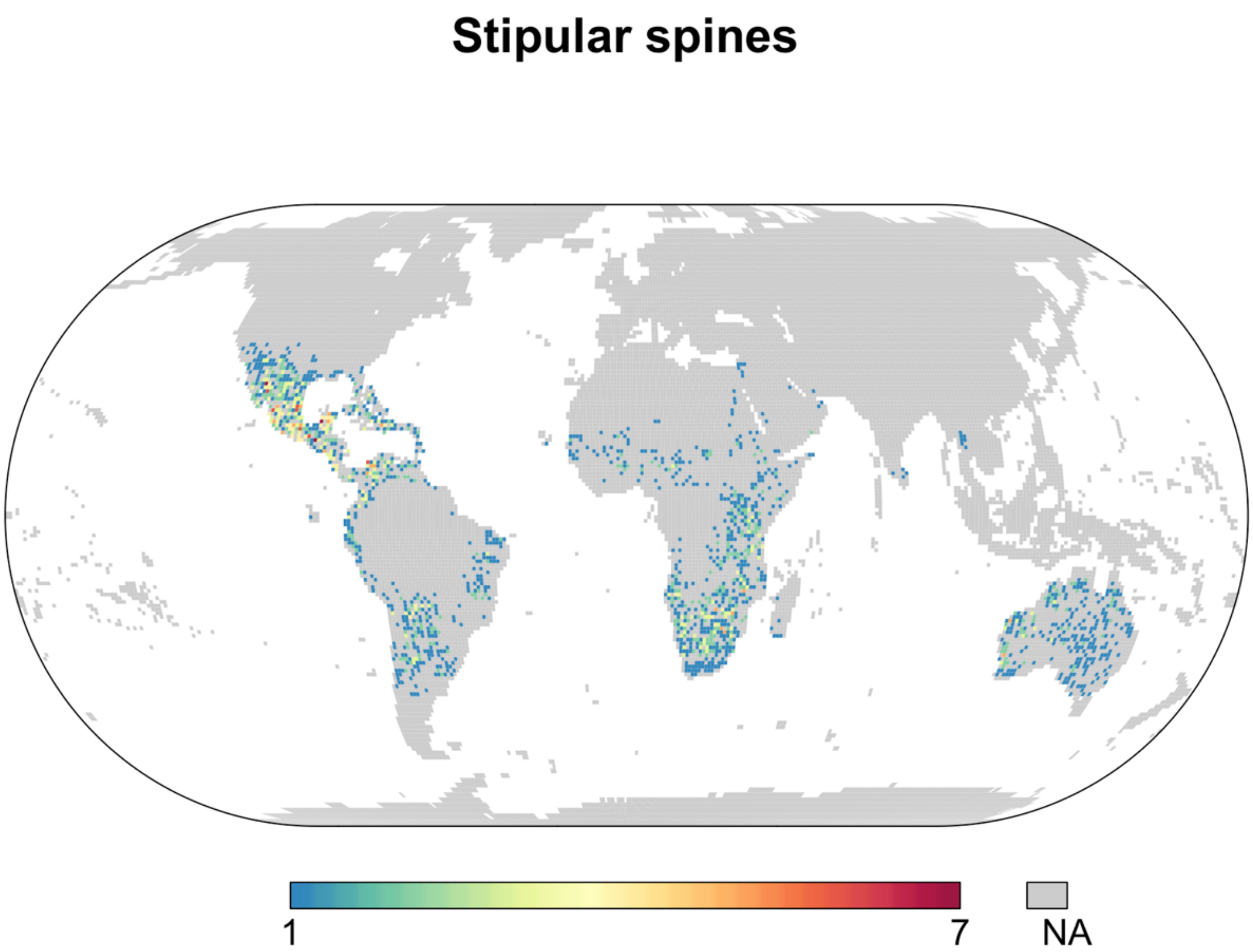
Stipular spines
1
7
NA

Figure S5: Association between plant growth form and spine type in Mimoseae.

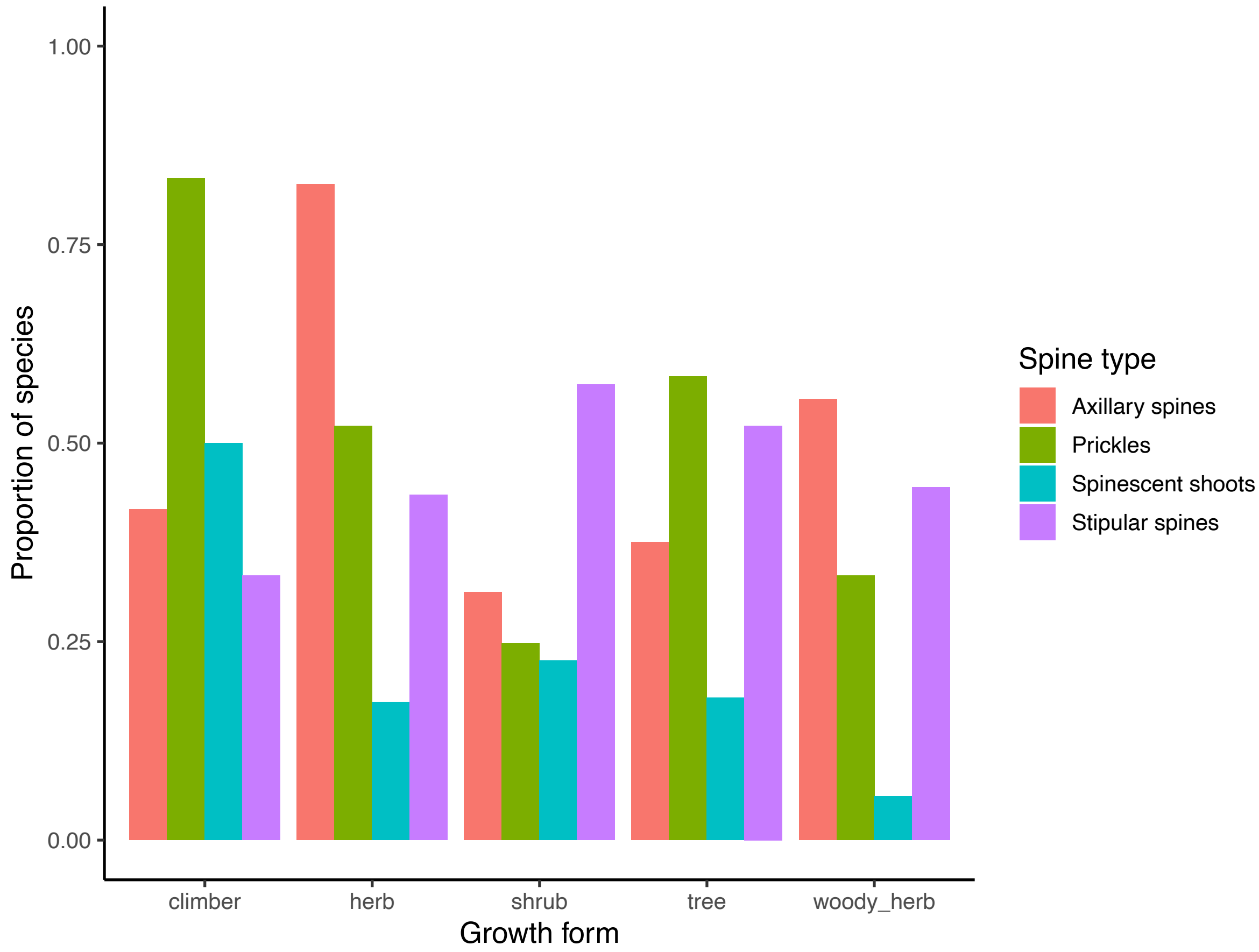

Notes S1: References used to score spinescence type per taxon.

African Plant Database. Conservatoire et Jardin botaniques de la Ville de Genève & South Afric. an National Biodiversity Institute, Pretoria. https://africanplantdatabase.ch (Accessed in 2022).

Aladesanmi, A. J. (2007). Tetrapleura tetraptera: Molluscicidal activity and chemical constituents. African Journal of Traditional, Complementary and Alternative Medicines, 4(1), 23–36.

Alves, F. M., Sartori, Â. L., Zucchi, M. I., Azevedo-Tozzi, A. M., Tambarussi, E. V., Alves-Pereira, A., & de Souza, A. P. (2018). Genetic structure of two Prosopis species in Chaco areas: A lack of allelic diversity diagnosis and insights into the allelic conservation of the affected species. Ecology and Evolution, 8(13), 6558–6574.

Arce, M. D. L. R., & Bachman, S. (2006). A taxonomic revision of Acaciella (Leguminosae, Mimosoideae). Anales del Jardín Botánico de Madrid, 63(2), 189–244.

Aronson, J. (1992). Evolutionary biology of Acacia caven (Leguminosae, Mimosoideae): Infraspecific variation in fruit and seed characters. Annals of the Missouri Botanical Garden, 79, 958–968.

Atahuachi, M. B., & Hughes, C. E. (2006). Two new species of Mimosa (Fabaceae) endemic to Bolivia. Brittonia, 58(1), 59–65.

Atlas of Living Australia. https://www.ala.org.au (Accessed in 2022, 2023 and 2024).

Bajpai, O., Srivastava, A. K., Kushwaha, A. K., & Chaudhary, L. B. (2014). Taxonomy of a monotypic genus Indopiptadenia (Leguminosae–Mimosoideae). Phytotaxa, 164(2), 61.

Barneby, R. C. (1991). Sensitivae censitae: A description of the genus Mimosa Linnaeus (Mimosaceae) in the New World. Memoirs of the New York Botanical Garden, 65.

Barneby, R. C. (1993). Increments to the genus Mimosa (Mimosaceae) from South America. Brittonia, 45(4), 328–332.

Barneby, R. C. (1997). Toward a census of genus Mimosa (Mimosaceae) in the Americas. Brittonia, 49(4), 452–457.

Barneby, R. C., & Fortunato, R. H. (1987). Four new diplostemonous species of Mimosa (Mimosaceae) from Paraguay and eastern Bolivia. Brittonia, 39(2), 165–174.

Barneby, R. C., & Grimes, J. W. (1984). Two new mimosaceous trees from the American tropics. Brittonia, 36(3), 236–240.

Barneby, R. C., & Grimes, J. W. (1997). Silk tree, guanacaste, monkey's earring: A generic system for the synandrous Mimosaceae of the Americas. Part II: Pithecellobium, Cojoba and Zygia. New York Botanical Garden.

Barros, M. J. F., & Morim, M. P. (2014). Senegalia (Leguminosae, Mimosoideae) from the Atlantic Domain, Brazil. Systematic Botany, 39(2), 452–477.

Bazante, M. L., Maas, P. J., & Barbosa, M. R. D. V. (2023). Duguetia leucotricha (Annonaceae), a new species from the Atlantic Coastal Forest of Brazil. Acta Botanica Brasilica, 37, e20220203.

Bentham, G. (1875). Revision of the suborder Mimoseae. Transactions of the Linnean Society of London, 30, 335–664.

Bocage, A. L. D., & Miotto, S. T. S. (2006). Acacia globosa and A. limae, two new Brazilian species. Rodriguésia, 57(1), 131–136.

Borges, L. M., Simon, M. F., & Pirani, J. R. (2017). Less is more: Adjusting the taxonomy of the polytypic Mimosa setosa. Rodriguésia, 68(2), 515–540.

Brenan, J. P. M. (1986). The genus Adenopodia (Leguminosae). Kew Bulletin, 73–90.

Britton, N. L., & Rose, J. N. (1928). Mimosaceae. In North American Flora (Vol. 23). New York Botanical Garden.

Burkart, A. (1939). Mimozyganthus, nuevo género de Leguminosas. Darwiniana, 3(3), 445–469.

Burkart, A. (1976). A monograph of the genus Prosopis. Journal of the Arnold Arboretum, 57, 450–525.

Capuron, R. (1969). Contributions à l'étude de la flore forestière de Madagascar: Sur la place du genre Kaliphora Hook. f. Adansonia, 9(3), 395–397. https://doi.org/10.5962/p.296577

Cialdella, A. M. (1984). El género Acacia (Leguminosae) en la Argentina. Darwiniana, 59–111.

Clokey, I. W. (1951). Flora of the Charleston Mountains, Clark County, Nevada. University of California Press.

Dash, S. S., & Sanjappa, M. (2011). Two new species and a new distributional record of Archidendron (Leguminosae: Mimosoideae) from India. Nelumbo, 7–16.

De Wit, H. C. D. (1952). A revision of the genus Archidendron F. Muell. (Mimosaceae). Reinwardtia, 2(1), 69–96.

Department of National Parks, Wildlife and Plant Conservation (DNP). e-Flora of Thailand. https://botany.dnp.go.th/eflora/ (Accessed in 2023).

Don, G. (1849). Niger flora: Or, an account of the plants of the interior of Africa. J. Ridgway.

Du Puy, D. (Ed.). (2002). The Leguminosae of Madagascar. Royal Botanic Gardens, Kew.

Dutra, V. F., & Garcia, F. C. P. (2014). Three new species of Mimosa sect. Mimosa from Minas Gerais, Brazil. Brittonia, 66(1), 33–41.

Dyer, C. (2014). New names for the African Acacia species in Vachellia and Senegalia. Southern Forests, 76(4), iii–iii.

Ebinger, J. E., Seigler, D. S., & Clarke, H. D. (2000). Taxonomic revision of South American species of the genus Acacia subgenus Acacia (Fabaceae: Mimosoideae). Systematic Botany, 25(4), 588-617.

Exell, A. W., & Wild, H. (1960). *Flora zambesiaca* (Vol. 1, Part I).

Federal Noxious Weed Disseminules of the United States. https://idtools.org/fnwd/ (Accessed in 2020).

Flora e Funga do Brasil (JBRJ). http://floradobrasil.jbrj.gov.br/ (Accessed in 2022 and 2023).

Flora Malesiana. https://floramalesiana.org/new/ (Accessed in 2022 and 2023).

Flora of Australia Online. Australian Biological Resources Study. http://www.environment.gov.au/science-research/abrs/online-resources/flora-of-australia-online (Accessed in 2022, 2023 and 2024).

Flora of China. http://www.efloras.org/flora_page.aspx?flora_id=2 (Accessed in 2022 and 2023).

Flora of North America. http://www.efloras.org/flora_page.aspx?flora_id= (Accessed in 2023).

Flora of Zimbabwe. https://www.zimbabweflora.co.zw (Accessed in 2022 and 2023).

Flores, E. M. (2002). Pentaclethra macroloba. In Tropical tree seed manual (Agricultural Handbook 721, pp. 601–604). USDA.

Forero, E., & Romero, C. (2005). Estudios en leguminosas colombianas.

Fortunato, R. H., & Palese, R. (1999). A new species of Mimosa from the Bolivian Chaco. Contribution to the study of the flora and vegetation of the Chaco XIII.

Fosberg, F. R. (1960). Serianthes Benth. (Leguminosae–Mimosoideae–Ingeae). Reinwardtia, 5(3), 293–317.

Gamble, J. S. (1920). The Indian species of *Mimosa*. *Bulletin of Miscellaneous Information (Royal Botanic Gardens, Kew)*, 1–6.

Grether, R. (2000). Nomenclatural changes in the genus *Mimosa*. *Novon*, 29–37.

Grether, R., & Martinez-Bernal, A. (1996). *Mimosa tejupilcana*, a new species. *Systematic Botany*, 617–621.

Grings, M., & Ribas, O. D. S. (2013). *Mimosa sobralii*, a new Brazilian tree species. *Phytotaxa, 131*(1), 23–28.

Guerra, E., Morim, M. P., & Iganci, J. R. V. (2016). A new species of *Abarema* from Brazil. *Phytotaxa, 289*(1), 77–82.

Hernandez, H. M. (1989). Systematics of *Zapoteca*. *Annals of the Missouri Botanical Garden*, 781–862.

Hernandez, H. M. (1990). A new subgenus and species of *Zapoteca*. *Systematic Botany*, 226–230.

Hernández, H. M., & Guinet, P. (1990). *Calliandropsis*, a new genus of Leguminosae: Mimosoideae from Mexico. *Kew Bulletin*, 609–620.

Hooker, W. J. (1852). *Journal of Botany: Being a Second Series of the Botanical Miscellany* (Vol. 4). Longman.

Hopkins, H. C. (1986). *Parkia* (Leguminosae: Mimosoideae). *Flora Neotropica*, 1–123.

Hughes, C. (1998). *Monograph of Leucaena (Leguminosae-Mimosoideae)* (Vol. 55, pp. 244-pp).

Hughes, C. E. (2016). Bolivian Mimosa (Leguminosae, Mimosoideae): three new species and a species checklist. *Phytotaxa*, *260*(3), 201-222.

Hughes, C. E., & B, M. A. (2006). A New Species of Leucochloron (Leguminosae: Mimosoideae) Endemic to Bolivia. Kew Bulletin, 559-563.

Iganci, J. R. V., & Morim, M. P. (2009). *Abarema* (Leguminosae, Mimosoideae) in the state of Rio de Janeiro, Brazil. *Rodriguésia, 60*(3), 581–594.

Iganci, J. R. V., & Morim, M. P. (2009). Three new species of *Abarema* (Leguminosae, Mimosoideae) from south-eastern Brazil. *Kew Bulletin, 64*(2), 271–277.

Kodela, P. G. (2001). *Acacia atrox* (Fabaceae: Mimosoideae), a new rare species from New South Wales. *Telopea, 9*(2), 415–419.

Ladyman, J. A. (2004). *Acacia greggii* Wildland Shrubs of the United States and Its Territories: Thamnic Descriptions: Volume, 16.

Lefèvre, G., & Labat, J. N. (2006). A new species of Mimosa (Fabaceae, Mimosoideae) from Madagascar. Novon: A Journal for Botanical Nomenclature, 16(1), 74-77.

Lewis, G. P., Hughes, C. E., Yomona, A. D., Sotuyo, J. S., & Simon, M. F. (2010). Three new legumes endemic to the Marañón Valley, Perú. Kew Bulletin, 65(2), 209-220.

Li, J. (2007). Flora of China. Harvard Papers in Botany, 13(2), 301-302.

Lim, T. K. (2011). Uvaria chamae. In Edible Medicinal and Non-Medicinal Plants: Volume 1, Fruits (pp. 231-236). Dordrecht: Springer Netherlands.

Linares, J. Especie nueva de Albizia (Leguminosae: Mimosoideae) de Centroamérica A new species of Albizia (Leguminosae: Mimosoideae) from Central America.

Lorence, D. H., & Wood, K. R. (1994). Kanaloa, a new genus of Fabaceae (Mimosoideae) from Hawaii. Novon, 137-145.

Luckow, M. (1993). Monograph of Desmanthus (leguminosae-mimosoideae). Systematic Botany Monographs, 1-166.

Mariod, A. A., Mirghani, M. E. S., & Hussein, I. H. (2017). Unconventional oilseeds and oil sources. Academic Press.

Maslin, B. R. (1980). Acacia (Leguminosae-Mimosoideae): A contribution to the flora of Central Australia. Journal of the Adelaide Botanic Garden, 301-321.

Maslin, B. R. (2014). Miscellaneous new species of Acacia (Fabaceae: Mimosoideae) from south-west Western Australia. Nuytsia, 24, 139-159.

Maslin, B. R., Barrett, M. D., & Barrett, R. L. (2013). A baker's dozen of new wattles highlights significant Acacia (Fabaceae: Mimosoideae) diversity and endemism in the north-west Kimberley region of Western Australia. Nuytsia, 23(1), 543-587.

Morales, M., & Fortunato, R. H. (2013). A new species of Mimosa (Mimosoideae, Leguminosae) from the inter-Andean dry valleys.

National Parks Board. Flora & Fauna Web. https://www.nparks.gov.sg/florafaunaweb (Accessed in 2023).

Nielsen, I. (1981). Notes on the genera Archidendron F. v. Mueller and Pithecellobium Martius in mainland SE Asia.

Nielsen, I. C. (1992). Flora Malesiana. Series I Á Spermatophyta, 11(part 1), 1Á226.

Nielsen, I., Guinet, P., & Baretta-Kuipers, T. (1983). Studies in the Malesian, Australian and Pacific Ingeae (Leguminosae-Mimosoideae): the genera Archidendropsis, Wallaceodendron, Paraserianthes, Pararchidendron and Serianthes (part I).

Nielsen, I., Prance, G. T., & Edmondson, J. R. (1983). Flore de la Nouvelle-Calédonie et Dépendances, volume 12: Legumineuses – Mimosoideae, Chrysobalanaceae, Plombaginaceae. Muséum national d'Histoire naturelle.

Oliver, D. (1868). Flora of tropical Africa (Vol. 1). L. Reeve and Company.

Orchard, A. E. (Ed.). (2001). Flora of Australia (Vol. 11). Csiro.

Pacific Island Ecosystems at Risk. http://www.hear.org/pier/ (Accessed in 2022)

Palacios, R. A. (2006). Los mezquites mexicanos: biodiversidad y distribución geográfica. Boletín de la Sociedad Argentina de Botánica, 41(1-2), 99-121.

Pedley, L. (1978). A revision of Acacia Mill. in Queensland. Austrobaileya, 75-234.

Pedley, L. (1999). Notes on Acacia (Leguminosae: Mimosoideae) chiefly from northern Australia. Austrobaileya, 307-321.

Pennington, T. D. (1997). The genus Inga: botany.

Plant Resources of the World (PROTA4U). https://prota.prota4u.org/ (Accessed in 2022).

Plants of the World Online. https://powo.science.kew.org/ (Accessed in 2021, 2022, 2023 and 2024).

Prain, D. (1898). Noviciæ Indicæ XV. Some additional Leguminosæ. Journal of the Asiatic Society of Bengal, Part II: Natural History, 66(2), 347.

de Queiroz, L. P., & Lewis, G. P. (1999). A new species of Mimosa from Bahia, Brazil. Kew Bulletin, 983–986.

Ribeiro, P. G. (2017). Filogenia molecular do grupo Piptadenia (Leguminosae, Caesalpinioideae, Clado Mimosoide) e taxonomia do gênero Piptadenia Benth.

Ribeiro, P. G., Luckow, M., Lewis, G. P., Simon, M. F., Cardoso, D., de Souza, É. R., ... & de Queiroz, L. P. (2018). Lachesiodendron, a new monospecific genus segregated from Piptadenia (Leguminosae: Caesalpinioideae: mimosoid clade): Evidence from morphology and molecules. *Taxon*, *67*(1), 37-54.

Rico-Arce, M. D. L. (1991). New species, combinations and synonyms for Zygia, Cojoba, marmaroxylon and Pithecellobium (Leguminosae: mimosoideae, Ingeae). *Kew Bulletin*, 493-521.

Ross, J. H. (1975). Notes on African Acacia species. *Bothalia*, *11*(4), 443-447.

Ross, J. H. (1975). The Acacia senegal complex. *Bothalia*, *11*(4), 453-462.

Roxburgh, W. (1819). Plants of the coast of Coromandel: Selected from drawings and descriptions presented to the Hon. Court of Directors of the East India Company. G. Nicol.

Särkinen, T. E., Marcelo-Peña, J. L., Yomona, A. D., Simon, M. F., Pennington, R. T., & Hughes, C. E. (2011). Underestimated endemic species diversity in the dry inter-Andean valley of the Río Marañón, northern Peru: An example from Mimosa (Leguminosae, Mimosoideae). *Taxon*, *60*(1), 139-150.

Sarwar, W. (2016). Pharmacological and phytochemical studies on Acacia modesta Wall; A review. *The Journal of Phytopharmacology*, *5*(4), 160-166.

Scalon, V. R. (2007). *Revisão taxonômica do gênero Stryphnodendron Mart.(leguminosae-mimosoideae)* (Doctoral dissertation, PhD thesis. Universidade de São Paulo).

Seigler, D. S., & Ebinger, J. E. (1988). Acacia macracantha, A. pennatula, and A. cochliacantha (Fabaceae: Mimosoideae) species complexes in Mexico. *Systematic Botany*, 7-15.

Seigler, D. S., Ebinger, J. E., & Miller, J. T. (2006). Mariosousa, a new segregate genus from Acacia sl (Fabaceae, Mimosoideae) from Central and North America. *Novon: A Journal for Botanical Nomenclature*, *16*(3), 413-420.

Seigler, D. S., Ebinger, J. E., Riggins, C. W., Terra, V., & Miller, J. T. (2017). Parasenegalia and Pseudosenegalia (Fabaceae): new genera of the Mimosoideae. *Novon: A Journal for Botanical Nomenclature*, *25*(2), 180-205.

Silva, Juliana Santos, and Margareth Ferreira de Sales. "Mimosa L.(Leguminosae-Mimosoideae) in the microrregion of Ipanema Valley, Pernambuco." Rodriguésia 59.3 (2008): 435-448.

Simon, M. F., Hughes, C. E., & Harris, S. A. (2010). Four new species of Mimosa (Leguminosae) from the central highlands of Brazil. *Systematic Botany*, *35*(2), 277-288.

Simon, M. F., Pastore, J. F. B., Souza, A. F., Borges, L. M., Scalon, V. R., Ribeiro, P. G., ... & Queiroz, L. P. (2016). Molecular phylogeny of Stryphnodendron (Mimosoideae, Leguminosae) and generic delimitations in the Piptadenia group. *International Journal of Plant Sciences*, *177*(1), 44-59.

South African National Biodiversity Institute. https://www.sanbi.org/ (Accessed in 2022 and 2023).

de Souza, É. R., Krishnaraj, M. V., & de Queiroz, L. P. (2016). Sanjappa, a new genus in the tribe Ingeae (Leguminosae: Mimosoideae) from India. Rheedea, 26(1), 1–12.

Ståhl, B., De L. Rico-Arce, M., & Lewis, G. P. (2010). Zygia nubigena sp. nov.(Leguminosae–Mimosoideae) from a submontane cloud forest in western Ecuador. Nordic Journal of Botany, 28(4), 453-456.

The New York Botanical Garden Herbarium (NY). https://sweetgum.nybg.org/science/vh/. (Accessed in 2022 and 2023).

Thulin, M. (2024). Studies of Asian 'Calliandra'lead to expansion of Sanjappa (Fabaceae-Caesalpinioideae). *Nordic Journal of Botany*, *2024*(1), e04241.

Turrill, W. B., & Milne-Redhead, E. (Eds.). (1953). Flora of tropical East Africa.

University of Arizona Campus Arboretum. (2022).

van Steenis, C. G. G. J. (1975). A review of the genus Sympetalandra Stapf and its position in Caesalpinioideae. *Blumea: Biodiversity, Evolution and Biogeography of Plants*, *22*(2), 159-167.

Vattakaven T, George R, Balasubramanian D, Réjou-Méchain M, Muthusankar G, Ramesh B, Prabhakar R (2016) India Biodiversity Portal: An integrated, interactive and participatory

biodiversity informatics platform. Biodiversity Data Journal 4: e10279. https://doi.org/10.3897/BDJ.4.e10279

Villiers, J. F. (1997). A New Species of Albizia Durazzini (Leguminosae Mimosoideae) from Madagascar. *Novon*, 420-422.

von Reis Altschul, S. (1964). A taxonomic study of the genus Anadenanthera. *Contributions from the Gray Herbarium of Harvard University*, (193), 3-65.

Warwick, M. C., & Lewis, G. P. (2003). Revision of Plathymenia (Leguminosae–Mimosoideae). *Edinburgh Journal of Botany*, *60*(2), 111-119.

Zich, F. A., Hyland, B. P. M., Whiffin, T., & Kerrigan, R. A. (2020). Australian tropical rainforest plants (Edition 8). https://apps.lucidcentral.org/rainforest/ (Accessed in 2022, 2023 and 2024).

Supplementary Tables:

Table S1: Glossary of morphological spinescence in Mimoseae. This table summarizes five categories of physical defence structures.

**Spinescence**	**Description**	**Origin**	**Reference**
Unarmed	No prickles, spines or thorns present	None	none
Stipular spines	Stipules modifies into rigid, pointed spines	Derived from modified stipules on each side of the leaf insertion	(Bell & Bryan, 2008)
Spinescent shoots	Lateral or terminal shoots or shoot segments modified into stiff, spine-like structures	Shoots (including short shoots) becoming spine-like	(Bell & Bryan, 2008)
Prickles	Sharp epidermal/cortical outgrowths lacking vascular tissue	Epidermal or sub-epidermal tissue	(Bell & Bryan, 2008)
Axillary spines	Accessory bud in the leaf axil that develops into a spine	Accessory buds located in the leaf axil	(Bell & Bryan, 2008)

Table S2: Extinct herbivore diet coverage by continent. Counts are numbers of species per continent (species occurring on multiple continents are counted in each). "Diet info" = species with a diet guild in HerbiTraits v1.2; "Mixed + browsers" = species classified as browsers or mixed-feeders (retained in analyses); "Grazers" = obligate grazers (excluded before SEMs); "Unknown" = no diet information (retained and flagged; none in this dataset).

Continent	Total species	Diet info	Mixed + browsers	Grazers	Unknown
Africa	35	35	26	9	0
America	81	81	71	10	0
Asia	42	42	31	11	0
Oceania	30	30	26	4	0

Table S3a: Standardized regression coefficients from the global Structural Equation Model (SEM) analyzing predictors (extant herbivory / dry season length) of spinescence in mimosoid legumes. At least 3 species of mimosoid per grid cell. Predictor variables include extant herbivory richness (*Extant*), dry season length (*lengthDry*) and mean annual temperature (*temp_chelsa*) and burn area (*BA*).

**Statistic**	**Value**
Estimator	ML
Optimization method	NLMINB
Number of parameters	10
Observations	3146
Chi-Square	0.642
CFI	1.000
TLI	1.008
RMSEA	0.000
• 90 % CI	0.000
• p (RMSEA ≤ 0.05)	1.000
• p (RMSEA ≥ 0.08)	0.000
SRMR	0.004
AIC	-8168.060
BIC	-8113.575
SABIC	-8124.172

**Outcome**	**Predictor**	**Estimate**	**Std.Err**	**z-value**	**p-value**	**Std.lv**	**Std.all**
Proportion spiny spp.	extant	0.634	0.021	16.511	0.000	0.345	0.280
	lengthDry	0.214	0.018	11.881	0.000	0.215	0.201
	temp_chelsa	-0.034					
extant	lengthDry	0.098	0.015	12.340	0.000	0.190	0.219
	temp_chelsa	-0.091	0.038	-3.307	0.001	-0.127	-0.059
	BA	0.389	0.096	4.474	0.000	0.454	0.083
BA	temp_chelsa	0.061	0.007	8.906	0.000	0.061	0.157

**R- Square**	**Estimate**
Proportion spiny spp.	0.190
extant	0.044
BA	0.025

Table S3a.1: Spatial error model for global spinescence (Extant herbivores + dry-season length).

	**Estimate**	**Std. Error**	**z value**	**Pr(>|z|)**
(Intercept)	0.063728	0.082979	0.768	0.4425
extant	0.027040	0.045295	0.597	0.5505
**lengthDry**	0.530064	0.021760	24.360	**<2e-16**

Table S3b: Standardized regression coefficients from the global Structural Equation Model (SEM) analyzing predictors (extinct herbivory / dry season length) of spinescence in mimosoid legumes. At least 3 species of mimosoid per grid cell. Predictor variables include extinct herbivore richness (Present Natural scenario, PN; *Extinct (PN)*), dry season length (*lengthDry*), mean annual temperature (*temp_chelsa*) and burn area (*BA*).

Statistic	Value
Estimator	ML
Optimization method	NLMINB
Number of parameters	11
Observations	3146
Chi-Square	0.965
CFI	1.000
TLI	1.007
RMSEA	0.015
• 90 % CI	0.000
• p (RMSEA ≤ 0.05)	0.999
• p (RMSEA ≥ 0.08)	0.000
SRMR	0.000
AIC	-9144.947
BIC	-9078.355
SABIC	-9113.306

Outcome	Predictor	Estimate	Std.Err	z-value	p-value	Std.lv	Std.all
Proportion spiny spp.	Extinct (PN)	0.432	0.024	17.859	0.000	0.432	0.322
	lengthDry	0.398	0.019	20.730	0.000	0.398	0.374
	temp_chelsa	0.113	0.046	2.468	0.014	0.113	0.043
Extinct (PN)	lengthDry	-0.284	0.013	-21.430	0.000	-0.284	-0.357
	temp_chelsa	-0.498	0.033	-15.064	0.000	-0.498	-0.254
	BA	0.987	0.082	11.974	0.000	0.987	0.196
BA	temp_chelsa	0.061	0.007	8.906	0.000	0.061	0.157

R- Square	Estimate
Proportion spiny spp.	0.156
Extinct (PN)	0.177
BA	0.025

Table S3b.1: Spatial error model for global spinescence (Extinct herbivores + dry-season length).

	Estimate	Std. Error	z value	Pr(>|z|)
(Intercept)	0.047045	0.088838	0.5296	0.596419
**Extinct (PN)**	0.090784	0.028390	3.1977	**0.001385**
**lengthDry**	0.540303	0.022004	24.5544	**< 2.2e-16**

Table S3c: Standardized regression coefficients from the global Structural Equation Model (SEM) analyzing predictors (extant herbivory / pH) of spinescence in mimosoid legumes. At least 3 species of mimosoid per grid cell. Predictor variables include extant herbivore richness (*Extant)* and soil pH **(*pH*)**, mean annual temperature (*temp_chelsa*) and burn area (*BA*).

**Statistic**	**Value**
Estimator	ML
Optimization method	NLMINB
Number of parameters	10
Observations	3141
Chi-Square	3.283
CFI	0.999
TLI	0.994
RMSEA	0.014
• 90 % CI	0.000
• p (RMSEA ≤ 0.05)	0.990
• p (RMSEA ≥ 0.08)	0.000
SRMR	0.008
AIC	-8374.578
BIC	-8314.055
SABIC	-8345.830

**Outcome**	**Predictor**	**Estimate**	**Std.Err**	**z-value**	**p-value**	**Std.lv**	**Std.all**
Proportion spiny spp.	Extant	0.616	0.020	15.550	0.000	0.317	0.258
	pH	0.432	0.024	17.608	0.000	0.421	0.292
Extant	pH	0.127	0.021	13.536	0.000	0.283	0.242
	temp_chelsa	-0.093	0.038	-2.992	0.000	-0.114	-0.054
	BA	0.442	0.096	5.981	0.000	-0.572	0.105
BA	temp_chelsa	0.052	0.007	7.350	0.000	0.052	0.133
	pH	-0.021	0.004	-5.397	0.000	-0.021	-0.098

**R- Square**	**Estimate**
Proportion spiny spp.	0.238
Extant	0.041
BA	0.034

Table S3c.1: Spatial error model for global spinescence (Extant herbivores + pH).

	**Estimate**	**Std. Error**	**z value**	**Pr(>|z|)**
(Intercept)	0.022147	0.068564	0.3230	0.7467
Extant	0.017467	0.044998	0.3882	0.6979
**pH**	0.731768	0.029076	25.1678	**<2e-16**

Table S3d: Standardized regression coefficients from the global Structural Equation Model (SEM) analyzing predictors (extinct herbivory / pH) of spinescence in mimosoid legumes. At least 3 species of mimosoid per grid cell. Predictor variables include extinct herbivore richness (*Extinct (PN)*)**,** and soil pH (*pH*)**,** mean annual temperature (*temp_chelsa*) and burn area (*BA*).

**Statistic**	**Value**
Estimator	ML
Optimization method	NLMINB
Number of parameters	11
Observations	3141
Chi-Square	0.623
CFI	1.000
TLI	1.000
RMSEA	0.000
• 90 % CI	0.000
• p (RMSEA ≤ 0.05)	0.965
• p (RMSEA ≥ 0.08)	0.000
SRMR	0.004
AIC	-10723.661
BIC	-10657.086
SABIC	-10692.038

**Outcome**	**Predictor**	**Estimate**	**Std.Err**	**z-value**	**p-value**	**Std.lv**	**Std.all**
Proportion spiny spp.	Extinct (PN)	0.542	0.025	28.930	0.000	0.725	0.484
	pH	0.760	0.025	33.422	0.000	0.821	0.570
	temp_chelsa	0.251	0.042	8.107	0.000	0.341	0.130
Extinct (PN)	pH	-0.446	0.016	26.754	0.000	0.417	-0.433
	temp_chelsa	-0.520	0.029	18.104	0.000	0.516	-0.294
	BA	0.787	0.071	9.161	0.000	0.654	0.145
BA	temp_chelsa	0.052	0.007	7.350	0.000	0.052	0.133
	pH	-0.021	0.004	-5.397	0.000	-0.021	-0.098

**R- Square**	**Estimate**
Proportion spiny spp.	0.257
Extinct (PN)	0.216
BA	0.034

Table S3d.1: Spatial error model for global spinescence (Extinct herbivores + pH).

	**Estimate**	**Std. Error**	**z value**	**Pr(>|z|)**
(Intercept)	-0.0060226	0.0735442	-0.0819	0.934734
**Extinct (PN)**	0.0861965	0.0281916	3.0575	**0.002232**
**pH**	0.7435061	0.0293285	25.3510	**< 2.2e-16**
temp_chelsa	0.0012765	0.0392216	0.0325	0.974036

Table S4a: Standardized regression coefficients from the Americas Structural Equation Model (SEM) analyzing predictors (extant herbivory / dry season) of spinescence in mimosoid legumes. At least 3 species of mimosoid per grid cell. Predictor variables include extant herbivore richness (*extant*), dry season length (*lengthDry*), mean annual temperature (*temp_chelsa*) and burn area (*BA*).

**Statistic**	**Value**
Estimator	ML
Optimization method	NLMINB
Number of parameters	11
Observations	1378
Chi-Square	0.016
CFI	0.996
TLI	0.962
RMSEA	0.059
• 90 % CI	0.109
• p (RMSEA ≤ 0.05)	0.286
• p (RMSEA ≥ 0.08)	0.291
SRMR	0.012
AIC	-9440.951
BIC	-9383.439
SABIC	-9418.382

**Outcome**	**Predictor**	**Estimate**	**Std.Err**	**z-value**	**p-value**	**Std.lv**	**Std.all**
Proportion spiny spp.	extant	0.357	0.130	2.752	0.006	0.357	0.057
	lengthDry	0.651	0.023	28.328	0.000	0.651	0.586
	temp_chelsa	-0.508	0.048	-10.666	0.000	-0.508	-0.217
extant	lengthDry	0.048	0.005	10.401	0.000	0.048	0.269
	temp_chelsa	-0.048	0.010	-4.883	0.000	-0.048	-0.129
	BA	0.148	0.025	-6.030	0.000	-0.148	-0.156
BA	lengthDry	0.021	0.005	-4.249	0.000	0.021	0.114
	temp_chelsa	0.093	0.011	8.851	0.000	0.093	0.237

**R- Square**	**Estimate**
Proportion spiny spp.	0.474
extant	0.132
BA	0.057

Table S4b: Standardized regression coefficients from the Americas Structural Equation Model (SEM) analyzing predictors (extinct herbivory / dry season) of spinescence in mimosoid legumes. At least 3 species of mimosoid per grid cell. Predictor variables include extinct herbivore richness (Present Natural scenario, PN; *Extinct (PN)),* dry season length (*lengthDry*), mean annual temperature (*temp_chelsa*) and burn area (*BA*).

Statistic	Value
Estimator	ML
Optimization method	NLMINB
Number of parameters	11
Observations	1378
Chi-Square	0.554
CFI	1.000
TLI	1.005
RMSEA	0.000
• 90 % CI	0.109
• p (RMSEA ≤ 0.05)	0.904
• p (RMSEA ≥ 0.08)	0.009
SRMR	0.003
AIC	-5237.459
BIC	-5179.947
SABIC	-5214.889

Outcome	Predictor	Estimate	Std.Err	z-value	p-value	Std.lv	Std.all
Proportion spiny spp.	Extinct (PN)	0.120	0.027	4.452	0.000	0.120	0.087
	lengthDry	0.673	0.022	30.358	0.000	0.673	0.607
	temp_chelsa	-0.530	0.047	-11.341	0.000	-0.530	-0.226
Extinct (PN)	lengthDry	-0.088	0.021	-4.141	0.04	-0.088	-0.109
	temp_chelsa	-0.131	0.046	-2.862	0.000	-0.131	-0.077
	BA	1.382	0.113	-12.214	0.000	1.382	-0.321
BA	lengthDry	0.021	0.005	4.249	0.000	0.021	0.114
	temp_chelsa	0.093	0.011	8.851	0.000	0.093	0.237

R- Square	Estimate
Proportion spiny spp.	0.478
Extinct (PN)	0.102
BA	0.057

Table S4c: Standardized regression coefficients from the Americas Structural Equation Model (SEM) analyzing predictors (extant herbivory / pH) of spinescence in mimosoid legumes. At least 3 species of mimosoid per grid cell. Predictor variables extant herbivore richness (*Extant*), soil pH (*pH*), mean annual temperature (*temp_chelsa*) and burn area (*BA*).

**Statistic**	**Value**
Estimator	ML
Optimization method	NLMINB
Number of parameters	11
Observations	1378
Chi-Square	0.513
CFI	1.000
TLI	1.001
RMSEA	0.000
• 90 % CI	0.109
• p (RMSEA ≤ 0.05)	0.985
• p (RMSEA ≥ 0.08)	0.000
SRMR	0.008
AIC	-10122.079
BIC	-10075.030
SABIC	-10103.620

**Outcome**	**Predictor**	**Estimate**	**Std.Err**	**z-value**	**p-value**	**Std.lv**	**Std.all**
Proportion spiny spp.	Extant	-0.819	0.120	-6.854	0.000	-0.819	-0.131
	pH	1.164	0.026	44.366	0.000	1.164	0.843
Extant	pH	0.107	0.005	20.753	0.000	0.107	0.483
	BA	-0.119	0.022	-5.423	0.000	-0.119	-0.126
BA	temp_chelsa	0.085	0.010	8.279	0.000	0.085	0.216

**R- Square**	**Estimate**
Proportion spiny spp.	0.621
Extant	0.261
BA	0.047

Table S4d: Standardized regression coefficients from the Americas Structural Equation Model (SEM) analyzing predictors (extinct herbivory / pH) of spinescence in mimosoid legumes. At least 3 species of mimosoid per grid cell. Predictor variables include extinct herbivore richness (*Extinct (PN)),* soil pH (*pH*)**,** mean annual temperature (*temp_chelsa*) and burn area (*BA*).

**Statistic**	**Value**
Estimator	ML
Optimization method	NLMINB
Number of parameters	10
Observations	1377
Chi-Square	0.676
CFI	1.000
TLI	1.003
RMSEA	0.000
• 90 % CI	0.109
• p (RMSEA ≤ 0.05)	0.982
• p (RMSEA ≥ 0.08)	0.000
SRMR	0.004
AIC	-5702.820
BIC	-5650.544
SABIC	-5682.310

**Outcome**	**Predictor**	**Estimate**	**Std.Err**	**z-value**	**p-value**	**Std.lv**	**Std.all**
Proportion spiny spp.	Extinct (PN)	0.166	0.024	6.920	0.000	0.166	0.120
	pH	1.095	0.023	47.775	0.000	1.095	0.793
Extinct (PN)	pH	-0.143	0.028	-5.047	0.000	-0.143	-0.143
	temp_chelsa	-0.190	0.049	-3.884	0.000	-0.190	-0.112
	BA	1.328	0.112	11.875	0.000	1.328	0.309
BA	temp_chelsa	0.085	0.010	8.192	0.000	0.085	0.215

**R- Square**	**Estimate**
Proportion spiny spp.	0.624
Extinct (PN)	0.108
BA	0.046

Table S5a: Standardized regression coefficients from the Africa Structural Equation Model (SEM) analyzing predictors (extant herbivory / dry season) of spinescence in mimosoid legumes. At least 3 species of mimosoid per grid cell. Predictor variables include extant herbivore richness (*extant*), dry season length (*lengthDry*), mean annual temperature (*temp_chelsa*) and burn area (*BA*).

Statistic	Value
Estimator	ML
Optimization method	NLMINB
Number of parameters	10
Observations	643
Chi-Square	0.056
CFI	0.995
TLI	0.956
RMSEA	0.061
• p (RMSEA ≤ 0.05)	0.261
• p (RMSEA ≥ 0.08)	0.453
SRMR	0.021
AIC	-1780.079
BIC	-1730.952
SABIC	-1765.876

Outcome	Predictor	Estimate	Std.Err	z-value	p-value	Std.lv	Std.all
Proportion spiny spp.	extant	0.256	0.060	4.262	0.000	0.256	0.139
	temp_chelsa	-0.203	0.113	-1.795	0.073	-0.203	-0.058
	lengthDry	0.987	0.048	20.645	0.000	0.987	0.617
extant	lengthDry	0.107	0.032	3.369	0.001	0.107	0.123
	temp_chelsa	-0.784	0.068	-11.571	0.000	-0.784	-0.411
	BA	0.228	0.103	2.208	0.027	0.228	0.081
BA	lengthDry	-0.072	0.012	-6.069	0.000	0.072	-0.233

R- Square	Estimate
Proportion spiny spp.	0.435
extant	0.191
BA	0.054

Table S5b: Standardized regression coefficients from the Africa Structural Equation Model (SEM) analyzing predictors (extinct herbivory / dry season) of spinescence in mimosoid legumes Predictor variables include extinct herbivore richness (Present Natural scenario, PN; *Extinct (PN)*), dry season length (*lengthDry*), mean annual temperature (*temp_chelsa*) and burn area (*BA*). Coefficients reflect direct paths only. All models were estimated using maximum likelihood with robust standard errors.

**Statistic**	**Value**
Estimator	ML
Optimization method	NLMINB
Number of parameters	9
Observations	643
Chi-Square	0.322
CFI	0.999
TLI	0.998
RMSEA	0.016
• p (RMSEA ≤ 0.05)	0.801
• p (RMSEA ≥ 0.08)	0.021
SRMR	0.015
AIC	-2866.019
BIC	-2825.824
SABIC	-2854.399

**Outcome**	**Predictor**	**Estimate**	**Std.Err**	**z-value**	**p-value**	**Std.lv**	**Std.all**
Proportion spiny spp.	Extinct (PN)	0.800	0.116	6.919	0.000	0.800	0.203
	lengthDry	0.999	0.047	21.264	0.000	0.999	0.607
Extinct (PN)	temp_chelsa	-0.469	0.030	-15.903	0.000	-0.469	-0.531
BA	lengthDry	-0.070	0.012	-5.963	0.000	-0.070	-0.228
	temp_chelsa	0.048	0.026	1.861	0.063	0.048	0.071

**R- Square**	**Estimate**
Proportion spiny spp.	0.442
Extinct (PN)	0.282
BA	0.059

Table S5c: Standardized regression coefficients from the Africa Structural Equation Model (SEM) analyzing predictors (extant herbivory / pH) of spinescence in mimosoid legumes Predictor variables include extant herbivore richness (*Extant*), soil pH (*pH),* mean annual temperature (*temp_chelsa*) and burn area (*BA*). Coefficients reflect direct paths only. All models were estimated using maximum likelihood with robust standard errors.

**Statistic**	**Value**
Estimator	ML
Optimization method	NLMINB
Number of parameters	11
Observations	643
Chi-Square	0.066
CFI	0.996
TLI	0.995
RMSEA	0.061
• p (RMSEA ≤ 0.05)	0.285
• p (RMSEA ≥ 0.08)	0.424
SRMR	0.013
AIC	-1852.377
BIC	-1803.250
SABIC	-1838.174

**Outcome**	**Predictor**	**Estimate**	**Std.Err**	**z-value**	**p-value**	**Std.lv**	**Std.all**
Proportion spiny spp.	Extant	0.253	0.057	4.429	0.000	0.253	0.138
	pH	1.247	0.054	23.228	0.000	1.247	0.658
	temp_chelsa	-0.385	0.108	-3.555	0.003	-0.385	-0.110
Extant	pH	0.115	0.038	3.011	0.000	0.115	0.112
	temp_chelsa	-0.801	0.068	-11.769	0.025	-0.801	-0.421
	BA	-0.235	0.105	2.241	0.000	-0.235	0.083
BA	pH	-0.101	0.014	-7.279	0.000	-0.101	-0.275
	temp_chelsa	0.061	0.025	2.385	0.017	0.061	0.090

**R- Square**	**Estimate**
Proportion spiny spp.	0.490
Extant	0.184
BA	0.083

Table S5d: Standardized regression coefficients from the Africa Structural Equation Model (SEM) analyzing predictors (extinct herbivory / pH) of spinescence in mimosoid legumes Predictor variables include extinct herbivore richness (*Extinct (PN)),* soil pH (*pH*), mean annual temperature (*temp_chelsa*) and burn area (*BA*). Coefficients reflect direct paths only. All models were estimated using maximum likelihood with robust standard errors.

**Statistic**	**Value**
Estimator	ML
Optimization method	NLMINB
Number of parameters	11
Observations	643
Chi-Square	0.079
CFI	0.997
TLI	0.973
RMSEA	0.057
• p (RMSEA ≤ 0.05)	0.313
• p (RMSEA ≥ 0.08)	0.394
SRMR	0.015
AIC	-2929.969
BIC	-2880.841
SABIC	-2915.765

**Outcome**	**Predictor**	**Estimate**	**Std.Err**	**z-value**	**p-value**	**Std.lv**	**Std.all**
Proportion spiny spp.	Extinct (PN)	0.622	0.132	4.719	0.000	0.622	0.157
	pH	1.250	0.052	23.325	0.000	1.250	0.658
	temp_chelsa	-0.292	0.116	-2.512	0.012	-0.292	-0.083
Extinct (PN)	pH	0.034	0.016	2.109	0.035	0.034	0.070
	temp_chelsa	-0.470	0.029	-15.980	0.000	-0.470	-0.532
BA	pH	-0.101	0.014	-7.279	0.000	-0.101	-0.275
	temp_chelsa	0.061	0.025	2.385	0.017	0.061	0.090

**R- Square**	**Estimate**
Proportion spiny spp.	0.491
Extinct (PN)	0.287
BA	0.083

Table S6a: Standardized regression coefficients from the global Structural Equation Model (SEM) analyzing predictors (extant herbivory / dry season) of spinescence in mimosoid legumes, with at least 1 mimosoid species per grid cell. Predictor variables include extant herbivore richness (*extant*), dry season length (*lengthDry*), mean annual temperature (*temp_chelsa*) and burn area (*BA*). Coefficients reflect direct paths only. All models were estimated using maximum likelihood with robust standard errors.

**Statistic**	**Value**
Estimator	ML
Optimization method	NLMINB
Number of parameters	9
Observations	5204
Chi-Square	0.406
CFI	1.000
TLI	1.000
RMSEA	0.000
• p (RMSEA ≤ 0.05)	1.000
• p (RMSEA ≥ 0.08)	0.000
SRMR	0.005
AIC	-16510.927
BIC	-16451.912
SABIC	-16480.511

**Outcome**	**Predictor**	**Estimate**	**Std.Err**	**z-value**	**p-value**	**Std.lv**	**Std.all**
Proportion spiny spp.	extant	0.628	0.028	22.741	0.000	0.628	0.286
	lengthDry	0.375	0.016	24.029	0.000	0.375	0.302
extant	lengthDry	0.108	0.008	13.837	0.000	0.108	0.192
	temp_chelsa	-0.051	0.018	-2.814	0.005	-0.051	-0.039
	BA	0.439	0.051	8.642	0.000	0.439	0.118
BA	temp_chelsa	0.048	0.005	10.188	0.000	0.048	0.140

**R- Square**	**Estimate**
Proportion spiny spp.	0.207
extant	0.053
BA	0.020

Table S6b: Standardized regression coefficients from the global Structural Equation Model (SEM) analyzing predictors (extinct herbivory / dry season) of spinescence in mimosoid legumes, with at least 1 mimosoid species per grid cell. Predictor variables include extinct herbivore richness (Present Natural scenario, PN; *Extinct (PN)),* dry season length *(lengthDry*), mean annual temperature (*temp_chelsa*) and burn area (*BA*). Coefficients reflect direct paths only. All models were estimated using maximum likelihood with robust standard errors.

**Statistic**	**Value**
Estimator	ML
Optimization method	NLMINB
Number of parameters	10
Observations	5204
Chi-Square	0.134
CFI	0.999
TLI	0.995
RMSEA	0.014
• p (RMSEA ≤ 0.05)	0.999
• p (RMSEA ≥ 0.08)	0.000
SRMR	0.007
AIC	-13959.352
BIC	-13893.780
SABIC	-13925.557

**Outcome**	**Predictor**	**Estimate**	**Std.Err**	**z-value**	**p-value**	**Std.lv**	**Std.all**
Proportion spiny spp.	Extinct (PN)	0.379	0.022	17.207	0.000	0.379	0.225
	lengthDry	0.516	0.016	31.292	0.000	0.516	0.416
	temp_chelsa	0.145	0.037	3.920	0.000	0.145	0.051
Extinct (PN)	lengthDry	-0.178	0.010	-18.120	0.000	-0.178	-0.242
	temp_chelsa	-0.345	0.023	-15.295	0.000	-0.345	-0.206
	BA	1.108	0.064	17.400	0.000	1.108	0.228
BA	temp_chelsa	0.048	0.005	10.188	0.000	0.048	0.140

**R- Square**	**Estimate**
Proportion spiny spp.	0.176
Extinct (PN)	0.121
BA	0.020

Table S6c: Standardized regression coefficients from the global Structural Equation Model (SEM) analyzing predictors (extant herbivory / pH) of spinescence in mimosoid legumes, with at least 1 mimosoid species per grid cell. Predictor variables include extant herbivore richness (*Extant*), soil pH (*pH*), mean annual temperature (*temp_chelsa*) and burn area (BA). Coefficients reflect direct paths only. All models were estimated using maximum likelihood with robust standard errors.

Statistic	Value
Estimator	ML
Optimization method	NLMINB
Number of parameters	10
Observations	5183
Chi-Square	0.001
CFI	0.995
TLI	0.956
RMSEA	0.043
• p (RMSEA ≤ 0.05)	0.640
• p (RMSEA ≥ 0.08)	0.006
SRMR	0.010
AIC	-16782.238
BIC	-16710.154
SABIC	-16745.108

Outcome	Predictor	Estimate	Std.Err	z-value	p-value	Std.lv	Std.all
Proportion spiny spp.	extant	0.627	0.027	23.491	0.000	0.627	0.286
	temp_chelsa	0.109	0.035	3.124	0.002	0.109	0.039
	pH	0.650	0.021	30.443	0.000	0.650	0.380
Extant	pH	0.137	0.011	12.453	0.000	0.137	0.175
	temp_chelsa	-0.055	0.018	-3.062	0.002	-0.055	-0.043
	BA	0.508	0.052	9.859	0.000	0.508	0.136
BA	temp_chelsa	0.038	0.005	7.920	0.000	0.038	0.111
	pH	-0.027	0.003	-9.171	0.000	-0.027	-0.129

R- Square	Estimate
Proportion spiny spp.	0.255
Extant	0.046
BA	0.036

Table S6d: Standardized regression coefficients from the global Structural Equation Model (SEM) analyzing predictors (extinct herbivory / pH) of spinescence in mimosoid legumes, with at least 1 mimosoid species per grid cell. At least 1 species of mimosoid per grid cell. Predictor variables include extinct herbivore richness (*Extinct (PN)*), soil pH (*pH),* mean annual temperature (*temp_chelsa*) and burn area (*BA*). Coefficients reflect direct paths only. All models were estimated using maximum likelihood with robust standard errors.

Statistic	Value
Estimator	ML
Optimization method	NLMINB
Number of parameters	11
Observations	5183
Chi-Square	0.083
CFI	0.999
TLI	0.993
RMSEA	0.020
• p (RMSEA ≤ 0.05)	0.969
• p (RMSEA ≥ 0.08)	0.000
SRMR	0.005
AIC	-14774.317
BIC	-14702.232
SABIC	-14737.186

Outcome	Predictor	Estimate	Std.Err	z-value	p-value	Std.lv	Std.all
Proportion spiny spp.	Extinct (PN)	0.515	0.022	23.737	0.000	0.515	0.305
	pH	0.910	0.022	40.546	0.000	0.910	0.532
	temp_chelsa	0.258	0.036	7.251	0.000	0.258	0.091
Extinct (PN)	pH	-0.330	0.013	-24.776	0.000	-0.330	-0.326
	temp_chelsa	-0.367	0.022	-16.731	0.000	-0.367	-0.220
	BA	0.911	0.063	14.549	0.000	0.911	0.188
BA	temp_chelsa	0.038	0.005	7.920	0.000	0.038	0.111
	pH	-0.027	0.003	-9.171	0.000	-0.027	-0.129

R- Square	Estimate
Proportion spiny spp.	0.256
Extinct (PN)	0.164
BA	0.036

Table S7a: Standardized regression coefficients from the global Structural Equation Model (SEM) analyzing predictors (extant herbivory / dry season) of spinescence in mimosoid legumes, with at least 5 mimosoid species per grid cell. Predictor variables include extant herbivore richness (*extant*), dry season length (*lengthDry*), mean annual temperature (*temp_chelsa*) and burn area (*BA*). Coefficients reflect direct paths only. All models were estimated using maximum likelihood with robust standard errors.

Statistic	Value
Estimator	ML
Optimization method	NLMINB
Number of parameters	9
Observations	2231
Chi-Square	0.535
CFI	1.000
TLI	1.004
RMSEA	0.000
• p (RMSEA ≤ 0.05)	0.999
• p (RMSEA ≥ 0.08)	0.000
SRMR	0.007
AIC	-8179.060
BIC	-8127.668
SABIC	-8156.262

Outcome	Predictor	Estimate	Std.Err	z-value	p-value	Std.lv	Std.all
Proportion spiny spp.	extant	0.611	0.033	18.299	0.000	0.611	0.360
	lengthDry	0.129	0.019	6.832	0.000	0.129	0.134
extant	lengthDry	0.092	0.012	7.554	0.000	0.092	0.163
	temp_chelsa	-0.078	0.030	-2.584	0.010	-0.078	-0.056
	BA	0.335	0.076	4.408	0.000	0.335	0.093
BA	temp_chelsa	0.064	0.008	7.977	0.000	0.064	0.167

R- Square	Estimate
Proportion spiny spp.	0.164
extant	0.040
BA	0.028

Table S7b: Standardized regression coefficients from the global Structural Equation Model (SEM) analyzing predictors (extinct herbivory / dry season) of spinescence in mimosoid legumes, with at least 5 mimosoid species per grid cell. Predictor variables include extinct herbivore richness (Present Natural scneario, PN; *Extinct (PN)),* dry season length (*lengthDry*), mean annual temperature (*temp_chelsa*) and burn area (*BA*). Coefficients reflect direct paths only. All models were estimated using maximum likelihood with robust standard errors.

**Statistic**	**Value**
Estimator	ML
Optimization method	NLMINB
Number of parameters	11
Observations	2231
Chi-Square	0.247
CFI	1.000
TLI	0.997
RMSEA	0.012
• p (RMSEA ≤ 0.05)	0.886
• p (RMSEA ≥ 0.08)	0.004
SRMR	0.007
AIC	-6988.775
BIC	-6925.963
SABIC	-6960.911

**Outcome**	**Predictor**	**Estimate**	**Std.Err**	**z-value**	**p-value**	**Std.lv**	**Std.all**
Proportion spiny spp.	Extinct (PN)	0.458	0.026	17.627	0.000	0.458	0.387
	lengthDry	0.336	0.021	15.845	0.000	0.336	0.351
	temp_chelsa	0.160	0.049	3.233	0.001	0.160	0.068
	lengthDry	-0.338	0.016	-21.403	0.000	-0.338	-0.417
	temp_chelsa	-0.551	0.039	-13.990	0.000	-0.551	-0.276
	BA	1.042	0.099	10.542	0.000	1.042	0.200
BA	temp_chelsa	0.064	0.008	7.977	0.000	0.064	0.167

**R- Square**	**Estimate**
Proportion spiny spp.	0.154
Extinct (PN)	0.217
BA	0.028

Table S7c: Standardized regression coefficients from the global Structural Equation Model (SEM) analyzing predictors (extant herbivory / pH) of spinescence in mimosoid legumes, with at least 5 mimosoid species per grid cell. Predictor variables include extant herbivore richness (*Extant*), soil pH (*pH*), mean annual temperature (*temp_chelsa*) and burn area (*BA*). Coefficients reflect direct paths only. All models were estimated using maximum likelihood with robust standard errors.

**Statistic**	**Value**
Estimator	ML
Optimization method	NLMINB
Number of parameters	10
Observations	2229
Chi-Square	0.649
CFI	1.000
TLI	1.008
RMSEA	0.000
• p (RMSEA ≤ 0.05)	0.996
• p (RMSEA ≥ 0.08)	0.000
SRMR	0.005
AIC	-8274.682
BIC	-8217.589
SABIC	-8249.360

**Outcome**	**Predictor**	**Estimate**	**Std.Err**	**z-value**	**p-value**	**Std.lv**	**Std.all**
Proportion spiny spp.	extant	0.587	0.033	18.017	0.000	0.587	0.345
	pH	0.306	0.025	12.417	0.000	0.306	0.238
Extant	pH	0.117	0.016	7.172	0.000	0.117	0.155
	temp_chelsa	-0.086	0.030	-2.830	0.005	-0.086	-0.062
	BA	0.388	0.076	5.079	0.000	0.388	0.107
BA	temp_chelsa	0.056	0.008	6.797	0.000	0.056	0.147
	pH	-0.015	0.005	-3.419	0.001	-0.015	-0.074

**R- Square**	**Estimate**
Proportion spiny spp.	0.202
Extant	0.038
BA	0.033

Table S7d: Standardized regression coefficients from the global Structural Equation Model (SEM) analyzing predictors (extinct herbivory / pH) of spinescence in mimosoid legumes, with at least 5 mimosoid species per grid cell. Predictor variables include extinct herbivore richness (*Extinct (PN)*), soil pH (*pH*), mean annual temperature (*temp_chelsa*) and burn area (*BA*). Coefficients reflect direct paths only. All models were estimated using maximum likelihood with robust standard errors.

Statistic	Value
Estimator	ML
Optimization method	NLMINB
Number of parameters	11
Observations	2229
Chi-Square	0.096
CFI	0.999
TLI	0.989
RMSEA	0.028
• p (RMSEA ≤ 0.05)	0.757
• p (RMSEA ≥ 0.08)	0.017
SRMR	0.008
AIC	-7403.947
BIC	-7341.145
SABIC	-7376.094

Outcome	Predictor	Estimate	Std.Err	z-value	p-value	Std.lv	Std.all
Proportion spiny spp.	Extinct (PN)	0.564	0.025	22.884	0.000	0.564	0.476
	pH	0.671	0.027	24.409	0.000	0.671	0.522
	temp_chelsa	0.291	0.046	6.273	0.000	0.291	0.123
Extinct (PN)	pH	-0.510	0.021	-24.713	0.000	-0.510	-0.470
	temp_chelsa	-0.559	0.038	-14.648	0.000	-0.559	-0.281
	BA	0.816	0.096	8.460	0.000	0.816	0.157
BA	temp_chelsa	0.056	0.008	6.797	0.000	0.056	0.147
	pH	-0.015	0.005	-3.419	0.001	-0.015	-0.074

R- Square	Estimate
Proportion spiny spp.	0.260
Extinct (PN)	0.256
BA	0.033

Table S8: Model selection for ancestral state reconstructions for different armature types under the ER and ARD models. This table presents the model fit for each armature type, scored as present / absent (Prickles, Stipular Spines, Axillary Spines, and Armed/Unarmed) under the Equal Rates (ER) and All Rates Different (ARD) models. AIC values for each model are provided to indicate the fit of the respective models.

Armature type	Model	logL	DF	AIC	Weight
Prickles	ER	-326.1293	1	654.2586	7.98e-22
**Prickles**	**ARD**	**-276.5494**	**2**	**557.0988**	**1.00**
Stipular spines	ER	-193.0008	1	388.0017	0.334
**Stipular spines**	**ARD**	**-191.3095**	**2**	**386.6190**	**0.666**
Axillary spines	ER	-16.76921	1	35.53843	0.0367
**Axillary spines**	**ARD**	**-12.50166**	**2**	**29.00333**	**0.9633**
Unarmed	ER	-462.1786	1	926.3572	0.00024
**Unarmed**	**ARD**	**-452.8337**	**2**	**909.6673**	**0.99976**
